# Ultrafast and accurate prediction of polycrystalline hafnium oxide phase-field ferroelectric hysteresis using graph neural networks†

**DOI:** 10.1039/d3na01115a

**Published:** 2024-04-02

**Authors:** Alhada-Lahbabi Kévin, Deleruyelle Damien, Gautier Brice

**Affiliations:** a INSA Lyon, Ecole Centrale de Lyon, CNRS, Universite Claude Bernard Lyon 1, CPE Lyon, INL, UMR5270 69622 Villeurbanne France kevin.alhada-lahbabi@insa-lyon.fr

## Abstract

Polycrystalline hafnium oxide emerges as a promising material for the future of nanoelectronic devices. While phase-field modeling stands as a primary choice tool for forecasting domain structure evolution and electromechanical properties of ferroelectric materials, it suffers from a high computational cost, which impedes its applicability to real-size systems. Here, we propose a Graph Neural Network (GNN) machine-learning framework to predict the ferroelectric hysteresis of polycrystalline hafnium oxide, with the goal of significantly accelerating computations in contrast to high-fidelity phase-field methods. By leveraging the inherent graph structure of the polycrystalline system and incorporating edge-level feature properties through graph attentional layers, our approach accurately predicts hysteresis behaviors across a broad range of polycrystalline structures, grain numbers, and Landau coefficients. The GNN framework exhibits high accuracy, with an average relative error of ∼4%, and demonstrates remarkable computational efficiency with respect to ground truth phase-field simulations, offering speed-ups exceeding a million-fold. Furthermore, we showcase the transferability of our model to efficiently scale predictions in polycrystals comprising up to a thousand grains, paving the way for effective simulations of real-sized systems. Our approach, by overcoming computational limitations in polycrystalline hafnium oxide, opens doors for accelerating discovery and design in ferroelectric materials.

## Abstract 2

Accelerate ferroelectric material discovery with a highly accurate and efficient Graph Neural Network predicting hysteresis in polycrystalline hafnium oxide.

## Graphical abstract

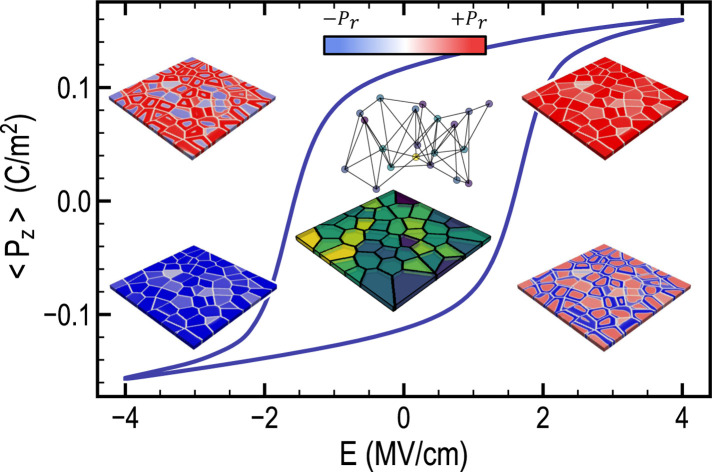

## Introduction

Ferroelectric thin films have recently gained substantial interest due to their potential applications in advanced electronic devices.^1^ The discovery of ferroelectricity in hafnium oxide (HZO) thin films^2–4^ has attracted considerable attention because of its high-performance electronic properties, scalability potential^5,6^ and compatibility with complementary metal oxide semiconductor (CMOS) technology.^5–8^ HZO-based electronic devices hold potential for a wide range of applications, including ferroelectric field-effect transistors (FeFETS), ferroelectric random access memories (FeRAM), negative capacitance devices, and ferroelectric tunnel junctions (FTJs).^7–10^

High-throughput phase-field simulations are commonly used to provide physical insight into ferroelectric materials.^11–13^ In polycrystalline PbTiO_3_-based systems, these simulations have significantly extended our understanding of the complex behavior arising from the interactions of polarization across grains and grain boundaries.^14–17^ In the past few years, the growing interest driven by the potential for HZO-based devices triggered a dramatic surge in efforts to understand domain dynamics in HZO polycrystalline thin films. While numerous phase-field approaches have been conducted to model the ferroelectric hysteresis properties of HZO,^18–23^ these endeavors did not always consider explicit representation of the grain and grain boundaries. Recent observations have underscored that precise delineation of the grain structure through Voronoi tessellation results in a noteworthy deviation within the simulated hysteresis.^24^ These findings revealed the pivotal role of accurately incorporating the crystalline arrangement in phase-field simulations.^24,25^

Furthermore, phase-field can also serve to establish correlations between experiments and simulations by calibrating material parameters. An example is the calibration of Landau coefficients, which has driven much interest due their fundamental significance in comprehending HZO properties.^19,24^ The resolution of those inverse problems involves optimizing the phase field inputs across the parameter space to match an experimental target. In a recent development, a genetic algorithm has been introduced to calibrate the Landau coefficients in three-dimensional polycrystalline HZO,^24^ employing the experimental ferroelectric hysteresis as a target to match during the phase-field simulations. While such approaches hold promise, they come with a high computational cost and may necessitate a trade-off between computational efficiency and physical accuracy, such as neglecting the depolarizing energy effect.^24^ In those parameter fine-tuning methods, this limitation is further accentuated by the necessity of iteratively repeating calculations, emphasizing the need for the acceleration of phase-field modeling.

With the objective to accelerate phase-field simulations, machine-learning approaches have been explored to build surrogate models.^13,26–37^ Many of these models are based on dense neural networks,^31,33^ recurrent neural networks,^29,32^ or convolutional neural networks (CNNs).^26–28,32^ CNNs typically operate on Euclidean data, conventionally utilizing the microstructure as input in the form of a 2D or 3D array. Learning from microstructure descriptors, they exhibit promising results,^26–28,32^ however, their efficiency is constrained when dealing with data that exhibits non-Euclidean topologies, such as polycrystalline arrangement. Specifically for polycrystalline materials, the use of geometric deep learning based on graph neural networks (GNNs) has proven to be highly effective in predicting material properties.^38–48^ GNNs enable the analysis of interactions between vertices *ν* (usually representing grains), which are connected by edges ℰ(typically representing grain boundaries). By incorporating node and edge features in the graph, GNNs can faithfully capture microstructural interactions, leading to more accurate results compared to CNN methods.^39^ Recently, Dai *et al.* introduced a GNN-based approach for predicting the effective magnetostriction of polycrystalline Tb_0.3_Dy_0.7_Fe_2_ computed by ferromagnetic phase-field.^39^ The microstructure was embedded in a graph, with node features including Euler angles (*α*, *β*, *γ*), grain voxels, and neighbor counts. Through graph convolutional network (GCN) message-passing layers and a fully connected layer, authors achieved accurate predictions of the effective magnetostriction for a single specific applied magnetic field.^39^ Notably, their model attained a low average error of ∼10%.^39^ While their model effectively replaces the ferromagnetic phase-field approach for predicting effective magnetostriction at at a given applied field, it does not encompass the broader the task of predicting the complete hysteresis, taking into account the impact of additional input parameters. Additionally, there have been notable advancements in achieving more accurate representations of material structures by leveraging edge-level feature properties,^38,44,48–50^ and employing graph attentional layers.^38^ The augmentation of physical information consistently yields enhanced graph representations, resulting in more efficient predictions of target properties.

In this paper, we present a machine-learning framework based on GNNs to tackle the computational challenges associated with phase-field simulations of ferroelectric hafnium oxide. The proposed model accurately predicts the complete ferroelectric hysteresis across a wide range of polycrystalline configurations, grain numbers, and Landau coefficients. By incorporating edge features and introducing graph attentional layers, we exploit the underlying graph structure to learn key nodes and edge-level interactions in polycrystalline ferroelectrics. Notably, our model achieves a relative error of ∼4%, underscoring its high accuracy and ability to capture the physical trends of polycrystal properties. The introduced framework predicts ferroelectric hysteresis at an exceptionally accelerated pace compared to classical phase-field, offering computational speed-ups exceeding a million-fold. Furthermore, an ablation study was carried out to quantify the influence of each architectural element in the GNN framework, thereby enhancing overall understandability. In this investigation, we also explored the utilization of transfer learning techniques to leverage insights gained from training GNNs on systems up to 150 nodes, enabling effective prediction of properties in real-size systems comprising up to a thousand grains. By overcoming computational limitations, our approach provides a viable pathway to facilitate the prediction of polycrystalline HZO ferroelectric properties, opening doors to accelerated materials discovery and design.

## Results and discussion

### Dataset generation

**Fig. 1:**
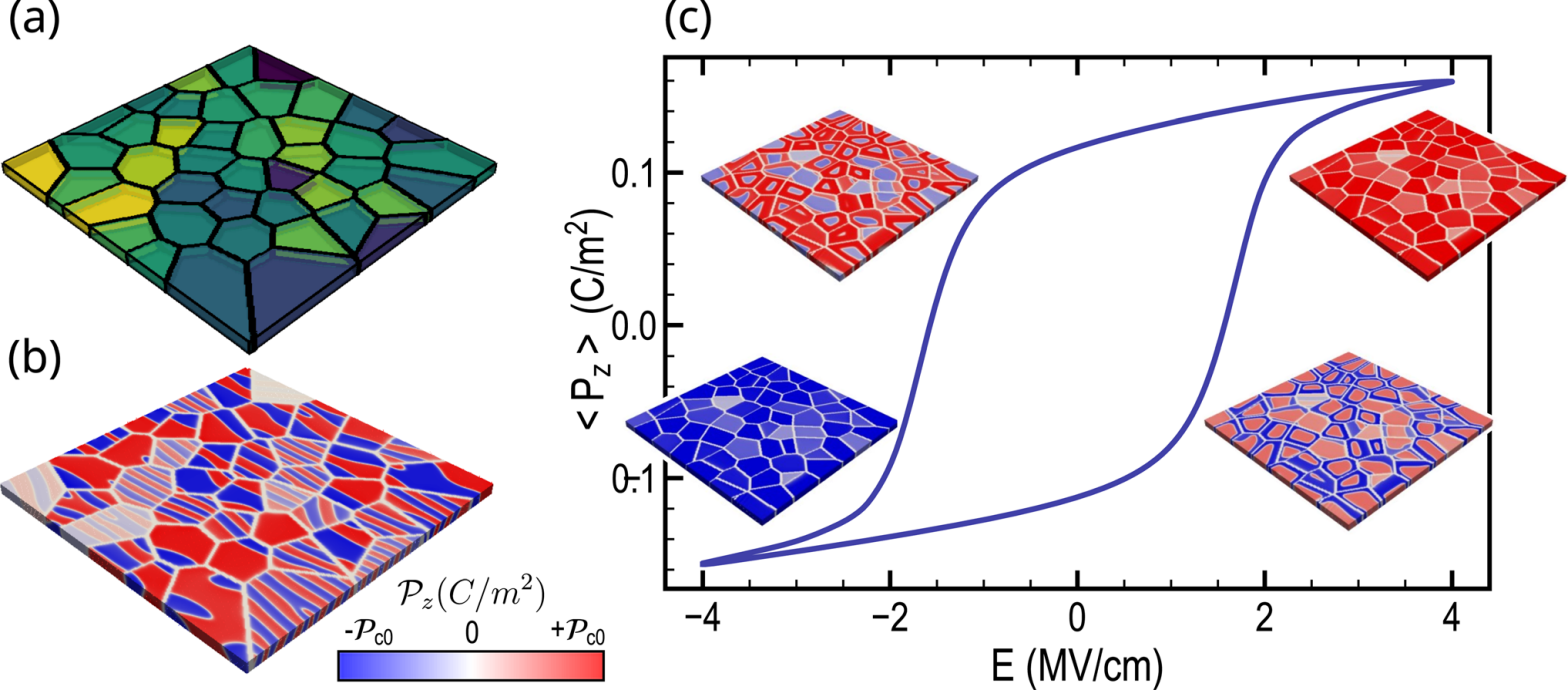
Phase-field simulation of polycrystalline hafnium oxide thin films. (a) Example of Voronoi structure from the dataset and (b) the corresponding polarization state at equilibrium in the absence of an applied voltage, revealing a complex domain structure. (c) Typical ferroelectric hysteresis obtained by phase-field on a 10 nm-thick polycrystalline thin film. The switching of ferroelectric grains is governed by their crystalline orientation and electrostatic interactions with neighboring grains.

This endeavor aims to develop a surrogate GNN model for predicting the ferroelectric hysteresis, utilizing the grain structures and Landau coefficients as inputs to the model. To create the dataset, we generated 3850 polycrystalline structures using Voronoi Tessellation (see Methods). Given the absence of consensus in the literature regarding the grain arrangement of HZO polycrystalline thin films,^24,25,51^ and in an effort to maintain a general approach, we have incorporated both columnar^24,52,53^ and equiaxed^24,54,55^ grains (Fig. 1a and S1†). Indeed, it has been experimentally observed that the intrinsic attributes of grains, whether they adopt a columnar configuration, traversing the complete thickness of the film,^24,52,53^ or exhibit an equiaxed morphology, distinguished by a succession of varied grains throughout the thickness,^24,54,55^ significantly impact the electrical characteristics of the sample.

In such polycrystalline thin films, a complex domain structure is commonly observed, characterized by various domains and domain walls within each grain. As an example, the domain state at equilibrium without applied voltage can be obtained through phase-field simulations, as illustrated in Fig. 1b. The ferroelectric hystereses were conducted using phase-field over 100 equal voltage steps from 4 to −4 volts. At each voltage step, the average out-of-plane ferroelectric polarization 〈*P*_z_〉 was recorded. A typical example of ferroelectric hysteresis obtained from phase-field simulation (see Methods) is depicted in Fig. 1c. The accompanying polarization evolution is depicted during domain switching, unveiling distinct switching dynamics for each grain based on its physical parameters and interactions with neighbors. Additional elucidation regarding the internal polycrystalline structure is provided through the 2D cross-sectional views presented in Fig. S2.† Furthermore, a detailed depiction of domain state evolution in the presence of grain and grain boundaries is given through 2D cross-sections and 3D views at equilibrium (Fig. S3 and S4†) and during ferroelectric hysteresis (Fig. S5†) within the ESI.† Specifically, Fig. S5† illustrates the interplay between grain orientation and the applied field, aligning with experimental observations that underscore the significance of accounting for polycrystallinity in HZO representation.^56^

**Fig. 2:**
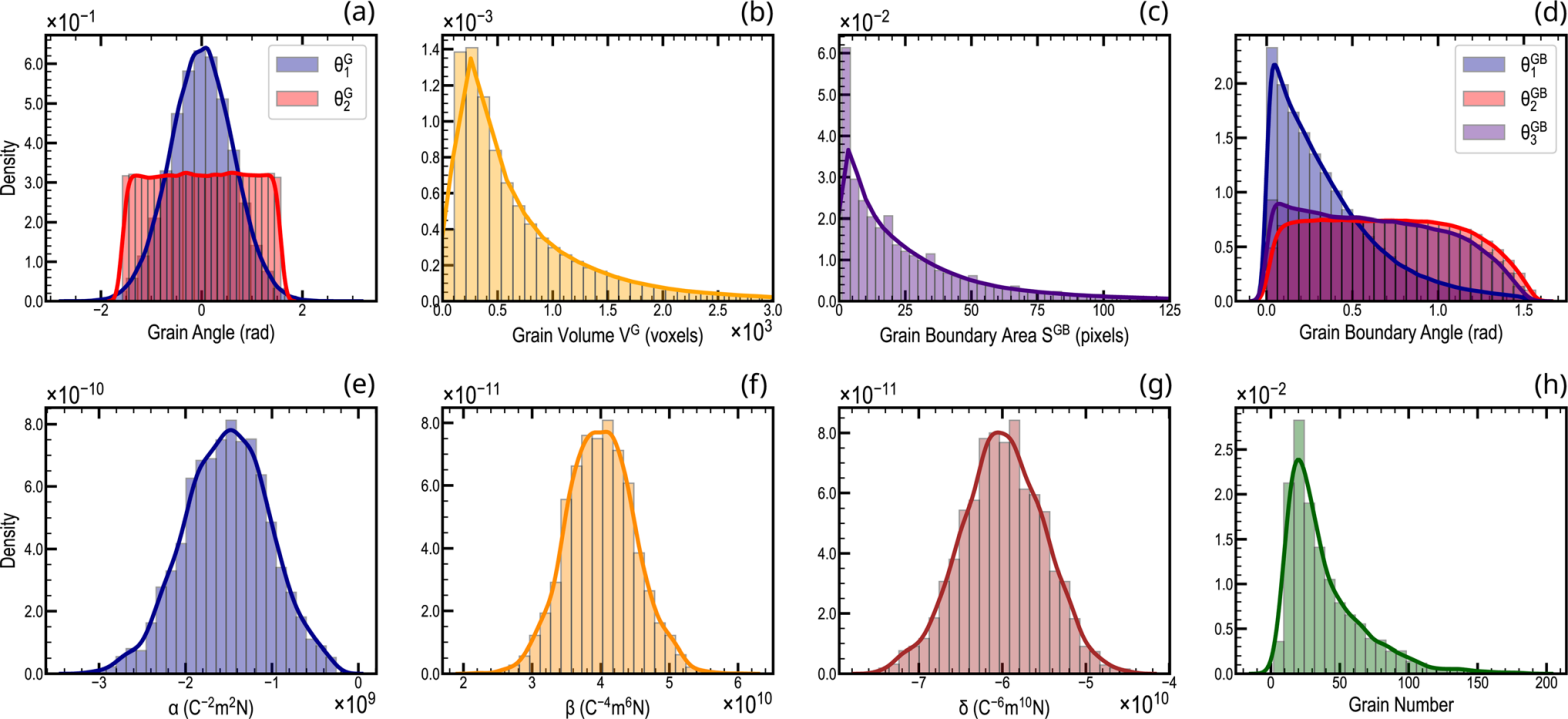
Data distribution across the 3D ferroelectric polycrystalline structures. (a) Angular distributions representing the orientation of the ferroelectric axis. Distribution of (b) grain volume, (c) grain boundary area, and (d) grain boundary orientation in the generated dataset. (e–g) Distribution of Landau coefficients (*α*, *β*, *δ*) covering a remanent polarization spectrum ranging from 5 to 20 μC cm^−2^. (h) The number of grains per structure varies broadly, ranging from 15 to 150.

An overview of the data distribution in the dataset used for this study is presented in Fig. 2. The orientation of ferroelectric grains in hafnium oxide has been recently reported in ref. 51, showing a predominant alignment along the out-of-plane direction. To capture the diverse orientations of ferroelectric axes in our dataset, each grain was randomly assigned an orientation of its ferroelectric axis (*θ*_1_^G^,*θ*_2_^G^) as shown in Fig. 2a. Specifically, we draw the angle *θ*_1_^G^ from a Gaussian distribution centered around 0 radians, reflecting the prevalent alignment trend observed in real samples where the orientation often aligns with the vertical direction. Simultaneously, *θ*_2_^G^ is drawn from a uniform distribution spanning values from 
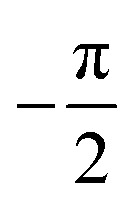
 to 
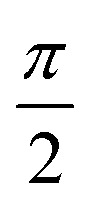
 radians (refer to the Methods section for details on the rotation matrix). By opting for this distribution, we ensure that our simulations cover the entire range of potential orientations, taking into consideration the inherent preference for out-of-plane alignment observed in actual hafnium oxide samples.^51,57,58^

In this work, we have opted to explore a spectrum of remanent polarization values around 15 μC cm^−2^, as a representative example commonly encountered in HZO experimental samples.^20,52,56,59–66^ To achieve this, Landau coefficients (*α*, *β*, *δ*) were randomly chosen from the Gaussian distributions shown in Fig. 2e–g. This results in a dataset with remanent polarization values ranging from 5 to 20 μC cm^−2^ and coercive fields from 1 to 3 MV cm^−1^, as exemplified in Fig. S6.† By varying the grain diameters from 7.5 to 20 nm, we ensured the dataset contains a wide polycrystalline structure variety. This choice was motivated by the experimental observation of the grain arrangement in polycrystalline samples, reporting average grain radius falling within a comparable range (∼13–16.6 nm (ref. 52), ∼5–20 nm (ref. 53), ∼10–20 nm (ref. 67), ∼5–15 nm (ref. 68)). Each polycrystal then roughly contains 15 to 150 ferroelectric grains as shown in Fig. 2h. The noteworthy variation in ferroelectric hysteresis arising from the heterogeneity of both the polycrystalline structures and Landau coefficients is illustrated in Fig. S7.†

Crucially, the range of remanent polarization values considered in our study encapsulates a diverse spectrum of experimental data denoted in the polycrystalline HZO literature.^52,56,59,61–66^ Specifically, the diverse shapes and remanent values observed in the ferroelectric hysteresis dataset (Fig. S7†) encapsulate a broad spectrum of experimental conditions, including variations in thermal annealing temperatures,^56,62,63,65^ wake-up processes,^56,59,62–64^ and polycrystalline morphology.^24,52,53,56,59,62–64,68^ Additionally, the range of coercive fields utilized to train the GNN framework is indicative of a substantial proportion of HZO samples fabricated with comparable thickness.^20,24,59,62,63,65,69^ The selection of Landau coefficients for dataset generation ensures that the (*P*_r_, *E*_c_) values also fall within the range consistent with simulation and experimental data encountered in various other phase-field analyses.^20,25,69^ Hence, the machine learning framework devised in this study is adept at capturing experimental data within a wide range of conditions. Further details regarding the influence of Voronoi structures and Landau coefficients on ferroelectric hysteresis are provided in Fig. S8 and S9.†

The resulting shape of each sample is then [(*G*,*α*,*β*,*δ*),(〈*P*_z_〉_0_,…,〈*P*_z_〉_100_)] where the graph *G* = (*V*, *E*) contains the nodes (*V*) and edges (*E*) of the polycrystalline system, (*α*, *β*, *δ*) being the Landau coefficients, and (〈*P*_z_〉_0_,…,〈*P*_z_〉_100_) the 100 points of each ferroelectric hysteresis. Finally, the dataset is split into a training dataset, which comprises 3150 structures to train the model, a validation dataset, which contains 350 structures, and a testing dataset of 350 structures.

### Graph structure

**Fig. 3:**
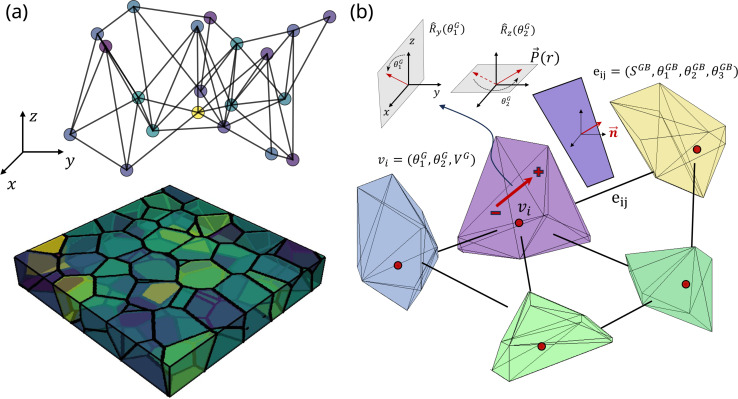
Graph embedding representation of the polycrystalline Voronoi structure. (a) A polycrystalline structure generated through Voronoi tessellation from the dataset and a connectivity representation. (b) Each ferroelectric grain *i* is represented by a node (*v*_*i*_) with node features including the orientation of the ferroelectric axis (*θ*_1_^G^,*θ*_2_^G^) and the grain volume *V*^G^. Two adjacent grains *i* and *j* are connected by an edge (*e*_*i,j*_), whose features consist of the area *S*^GB^ and orientations of the contact plane (*θ*_1_^GB^, *θ*_2_^GB^, *θ*_3_^GB^).

In this endeavor, our primary objective is to directly predict the complete ferroelectric hysteresis of polycrystalline hafnium oxide thin films. The hysteresis curve comprises 100 Polarization–Voltage (PV) points and is predicted by utilizing information extracted from the crystalline structure and the Landau coefficients as inputs to the machine learning framework. To this end, the grain structure is embedded in a graph, where the ferroelectric grains are represented as the nodes of the graph.^39^ The connectivity between neighboring nodes is determined by an adjacency matrix, denoted as *A* (see Methods). An example of Voronoi structure and a connected graph is given in Fig. 3a. To fully leverage the potential of GNNs, the physical embedding must reflect the physics governing domain-switching in ferroelectric polycrystals. This requires a careful consideration of the relevant physical node and edge features to be accounted. At the node level, we included the two angles (*θ*_1_^G^,*θ*_2_^G^) that indicate the polarization orientation inside each grain, as well as the voxel volume *V*^G^ of the grains, as shown in Fig. 3b. Since the electrostatic equilibrium is accounted through the resolution of Poisson equation in the phase-field simulations (see Methods), the grains electrically interact during domain switching through the polarization charge created at each grain boundary interface. Electrostatic coupling between two grains is considerably influenced by the orientations of their polarizations and grain boundary planes. Consequently, we introduced edge-level features, specifically focusing on grain boundary area denoted as *S*_GB_, along with the three angles (*θ*_1_^GB^,*θ*_2_^GB^,*θ*_3_^GB^) that describe the orientations of the grain boundaries, as represented in Fig. 3b.

### Graph architecture

In graph neural networks, information is exchanged between nodes through message-passing layers (MPLs).^70,71^ Since our approach emphasizes learning grain interaction from node and edge features, we chose to use graph attentional layers (GATs) (see Methods).^71^ These layers allow for the consideration of edge-level information in the overall graph representation.

**Fig. 4:**
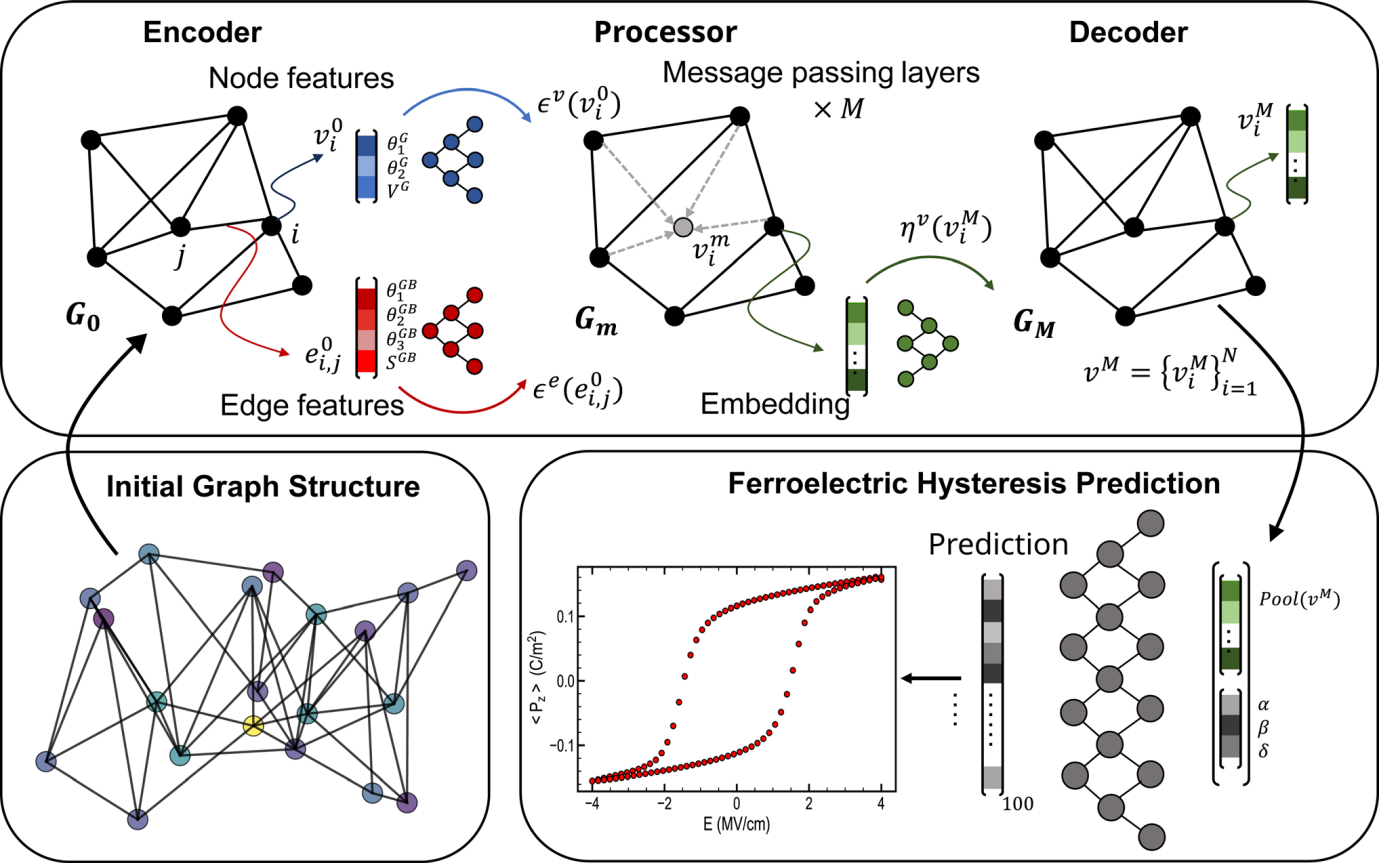
Architecture of the GNN framework. The encoder embeds the initial graph representation into a deeper latent space before several graph-attentional MPLs are applied to the graph structure. Subsequently, the decoder extracts the information from each node. A pooling layer is then used to gather information from all nodes. Finally, this embedding is concatenated to the Landau coefficients before a dense neural network outputs the 100 points constituting the hysteresis.

In order to improve the expressivity of our GNN framework, we adopted an encoder-processor-decoder architecture as in other graph-based studies,^72,73^ described in Fig. 4. The graph representation is denoted *G* = (*ν*,ℰ) with N nodes (*v*_*i*_ ∈ *ν*), and edges (*e*_*i,j*_ ∈ ℰ). The initial grain-based representation *G*^0^ is constructed by embedding each ferroelectric grain's nodes and edges.

The encoder is implemented as two multi-layer perceptrons (MLPs) *ε*^v^ and *ε*^e^. They embed the initial nodes and edges states into the latent vectors *v*_*i*_^1^ = *ε*^*v*^(*v*_*i*_^0^) and *e*_*i,j*_^1^ = *ε*^e^(*e*_*i,j*_^0^). After encoding, the embedded representation *G*^1^ = (*v*_*i*_^1^,*e*_*i,j*_^1^) serves as the input graph for the processor. The processor consists of *M* steps of message-passing layers built upon learnable graph attentional layers *G*^*m*^ = MPL^*m*^(*G*^*m* − 1^) (*m* = 1, …, *M*). This process successively updates the graph's latent representation, enabling information propagation deeper into the graph. Ultimately, the processor outputs a final graph *G*^*M*^ = MPL^*M*^(…MPL^1^(*G*^1^)), with node representations *v*_*i*_^M^. Afterward, the decoder *η*, represented as an MLP, extracts information from the final node representations *v*_*i*_^M^ of the graph, aiming to convert them into the node outputs *y*_*i*_ = *η*(*v*_*i*_^M^), relevant to the hysteresis prediction task. To derive graph-level outputs from the node information, a global mean pool layer is then employed to average node features across node dimensions.^74^ Following this, the resulting embedding is concatenated with the Landau coefficients (*α*, *β*, *δ*), and a final multilayer perceptron is used to produce ferroelectric hysteresis predictions.

### Hysteresis prediction

**Fig. 5:**
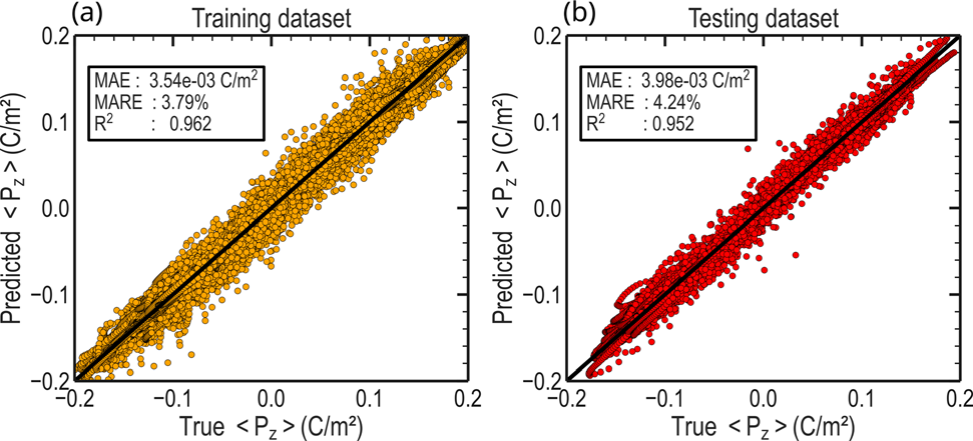
Evaluation of the model prediction. Out-of-plance polarization 〈*P*_z_〉 extracted from the GNN framework predictions on the (a) training and (b) testing datasets. The mean absolute error (MAE), macro average relative error (MARE), and the coefficient of determination *R*^2^ are reported to evaluate model performances for both datasets.

To assess the model's performance after training, we used the mean absolute error (MAE), the macro average relative error (MARE), and the coefficient of determination *R*^2^. Further details regarding the training, loss function, and metrics computation can be found in the Methods section. To report statistically meaningful results, we initialized and trained 50 models independently, all sharing the same architecture. We show here the results for the best model in Fig. 5, for both the training set of 3150 structures and the testing set of 350 structures. The model exhibits a low MARE of 3.79% and 4.24% on the training and testing datasets, respectively. For the respective datasets, the MAE is 3.54 × 10^−3^ C m^−2^ and 3.98 × 10^−3^ C m^−2^. These errors remain very small and have negligible impact on the physical interpretation of the results when compared to the spontaneous polarization's order of magnitude. The surrogate model consistently reproduces the physical simulator prediction, as evidenced by the high correlation coefficient *R*^2^ = 0.952 when comparing machine learning and phase-field outcomes, as presented in Fig. 5. With a low error on both datasets, the GNN framework shows good generalization from learning and does not exhibit overfitting. Remarkably, the model demonstrates great stability to randomness during the training, since the average MARE computed on the testing dataset for the 50 trained independent models is below 5%. It confirms that the model was able to learn the underlying concepts of phase-field and is capable of accurately predicting the hysteresis behavior of ferroelectric polycrystals.

**Fig. 6:**
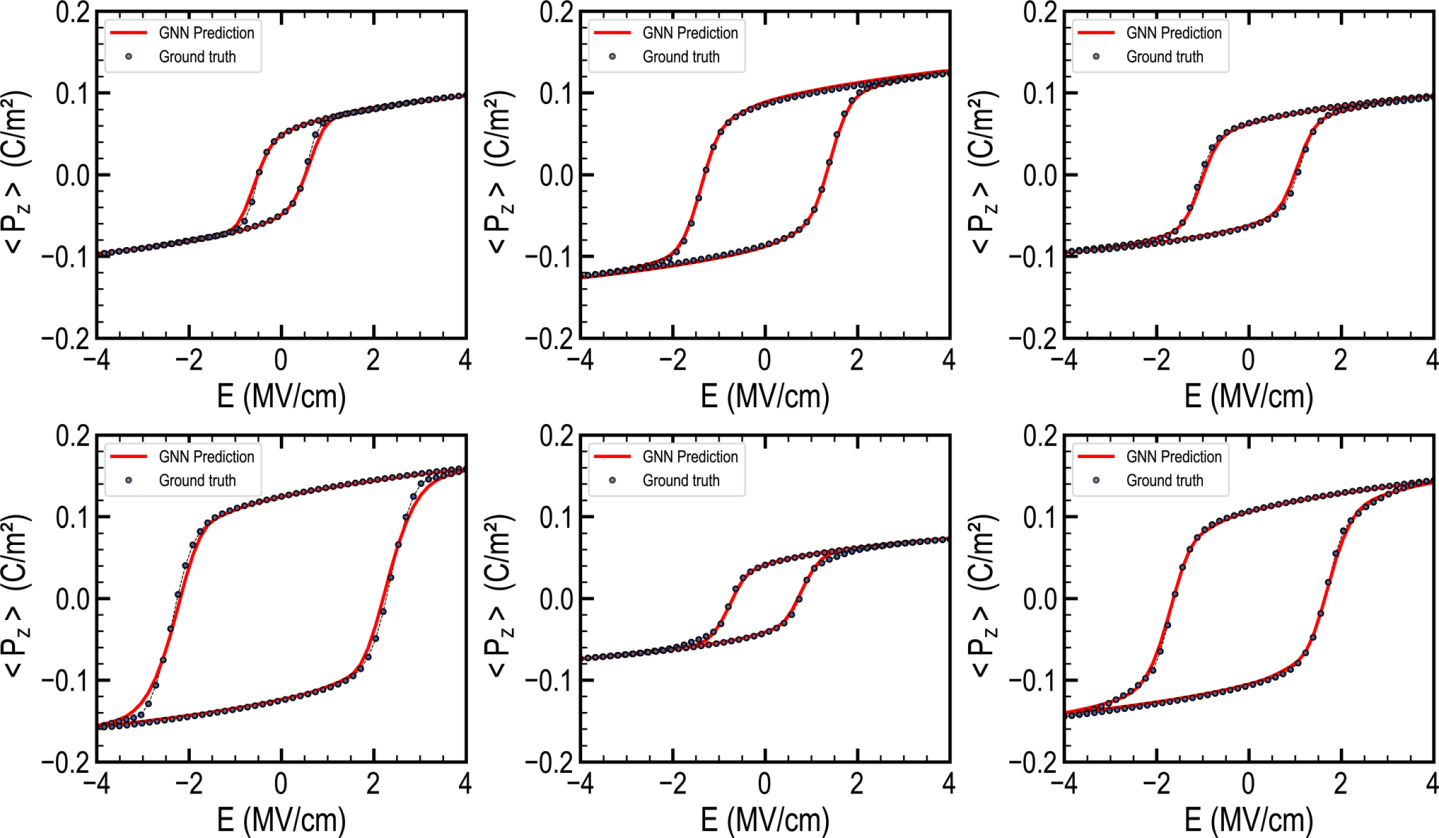
Ferroelectric hysteresis prediction by the GNN framework. Illustrations of ferroelectric hysteresis predictions are presented alongside their corresponding ground truths from the testing dataset. The model predictions are displayed with solid lines for the sake of clarity, despite the fact that they are composed of 100 discrete points. The ground truths are represented by discrete data points. The model's predictions accurately capture various types of hysteresis shapes.

Fig. 6 displays several instances of hysteresis taken from the testing dataset alongside the corresponding predictions made by the GNN model. The ferroelectric hysteresis outputs produced by our framework exhibit a remarkable agreement with the ground truths. Our model faithfully captures the hysteresis physical trends, reproducing crucial parameters such as remanent polarization, saturation polarization, and coercive fields. Notably, the predictions displayed in Fig. 6 cover a large array of hysteresis shapes, evidencing the model's ability to accurately represent a wide range of material behavior.

### Computational efficiency

An important challenge in using phase-field modeling for inverse problems, calibrating physical parameters, or conducting large-scale statistical analyses lies in the extensive computational time. Particularly in 3D simulations, solving partial differential equations for electrostatic and mechanical equilibrium demands considerable computational resources, often necessitating compromises in resolving these equations.^24^

To assess the acceleration achieved by the GNN framework, we computed the time required to predict the 3500 ferroelectric hystereses in the training and validation dataset. As a point of comparison, it took ∼175 hours (∼7 days) to generate the predictions by phase-field with an INTEL i9 CPU clocked at 5.1 GHz, while it took less than 0.2 seconds for the GNN to perform the same predictions using a GPU NVIDIA GeForce RTX3080. This remarkable acceleration led to an average inference time of 60 μs per prediction, providing an acceleration of 3.15 × 10^6^. Although GNNs are designed to take full advantage of GPU, we also computed the GNN inference time using the INTEL i9 CPU to provide fair comparisons on the same material. Using the surrogate model with the CPU, the prediction for all 3500 simulations took ∼1.2 seconds, yielding an inference time of 0.34 ms per hysteresis and an acceleration factor of 525 000. Even though our GNN exhibits ultrafast inference times, our approach also entails the time cost required for both training and generating the dataset. Training the model took approximately 15 minutes. This duration for neural network training remains very short. However, the primary time-consuming aspect currently lies in generating the datasets, which takes around 7 days due to the inherent runtime constraints of phase-field simulations. While the generation of datasets represents a significant initial time commitment, it is crucial to emphasize that this phase is a one-time expenditure. Following this initial investment, the subsequent use and application of the GNN model incur minimal time costs, underscoring the long-term effectiveness of our approach. Moreover, the potential for model scalability to other systems through transfer learning, combined with the requirement to generate only a limited amount of new data, further highlights the efficiency and adaptability of our approach.

### Ablation study

With the objective of comprehensively assessing the contributions of different components within the framework and to gain deeper insights into the learning process, we conducted ablation studies involving the training dataset size and model architecture.

**Fig. 7:**
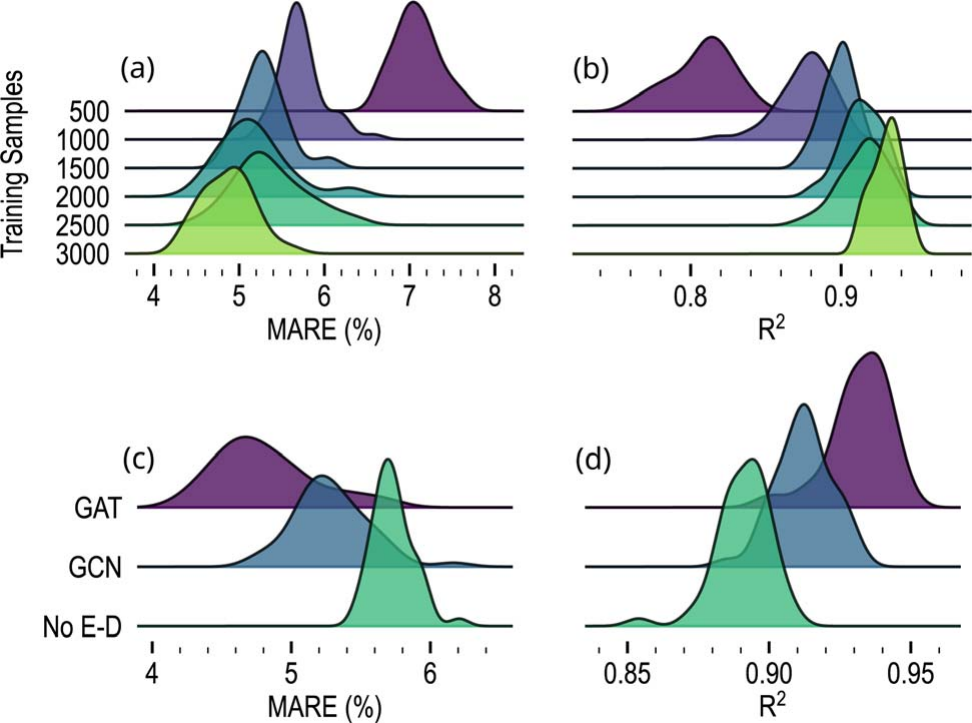
Ridgeline plots of performance comparisons across different numbers of polycrystalline structures in the training dataset and architecture choices. The top plots illustrate the results regarding the training dataset size, measured in terms of (a) MARE and (b) *R*^2^ for datasets containing 500, 1000, 1500, 2000, 2500, and 3000 structures. The bottom plots illustrate the (c) MARE and (d) *R*^2^ scores for three model configurations: the framework outlined in this paper (GAT), an alternative employing Graph Convolutional Network message passing layers (GCN), and a model omitting the encoder and decoder components (No E-D). Results are sorted by lowest MARE and *R*^2^ scores across each prediction task.

To evaluate the importance of the size of the training dataset on model performance, we trained the model with different numbers of training samples. We used the same hyperparameters as detailed in the Methods section, and the MARE and *R*^2^ scores were computed on the 350 structures of the testing dataset. The training dataset was progressively reduced from 3000 down to 500 structures. To ensure statistical significance, we trained and evaluated 50 models for each dataset size. The distribution of MARE and *R*^2^ of these 50 models on the validation dataset is reported in Fig. 7a and b for each training size. The model's performance exhibits a noticeable enhancement as the number of training samples increases, underscoring the benefit of a larger dataset for learning. Significant improvements are observed as the training dataset increases from 500 to 1500 structures. The MARE increased from 5% to 7%, and the coefficient of determination increased from roughly 0.8 to 0.9. Afterwards, the further expansion of the dataset to 3000 samples yields decaying improvements in performance.

In order to elucidate the significance of Message-Passing Layers (MPLs) and the information exchanged during message-passing steps, we replaced the Graph Attentional Layers (GATs) with Graph Convolutional Layers (GCNs) where edge information is not considered in node interactions (see Methods). For both architectures, 50 models were trained using the complete training dataset of 3150 structures. Fig. 7c and d depict the MARE and *R*^2^ results for each model. The model architecture using GATs achieved the highest scores. Despite yielding slightly inferior results, the framework that employs GCNs still produces accurate predictions, with an average MARE just above 5% and an *R*^2^ score of 0.92. Furthermore, we conducted an additional ablation study to assess the enhancement introduced by the encoder and decoder. To achieve this, we removed these networks from the framework while keeping the other hyperparameters unchanged and utilizing graph attentional layers as the MPLs. The resulting scores exhibited a slight decrease, with a MARE above 5.5% and a correlation coefficient lower than 0.90, as demonstrated in Fig. 7c and d. This finding underscores the importance of choosing appropriate message-passing layers and the value of incorporating the encoder and decoder in enhancing model performance.

### Scaling graph neural networks for larger systems

To perform reliable phase-field modeling of HZO thin films, it is essential to simulate a large number of grains, up to several hundreds,^24^ which presents considerable computational limitations.

**Fig. 8:**
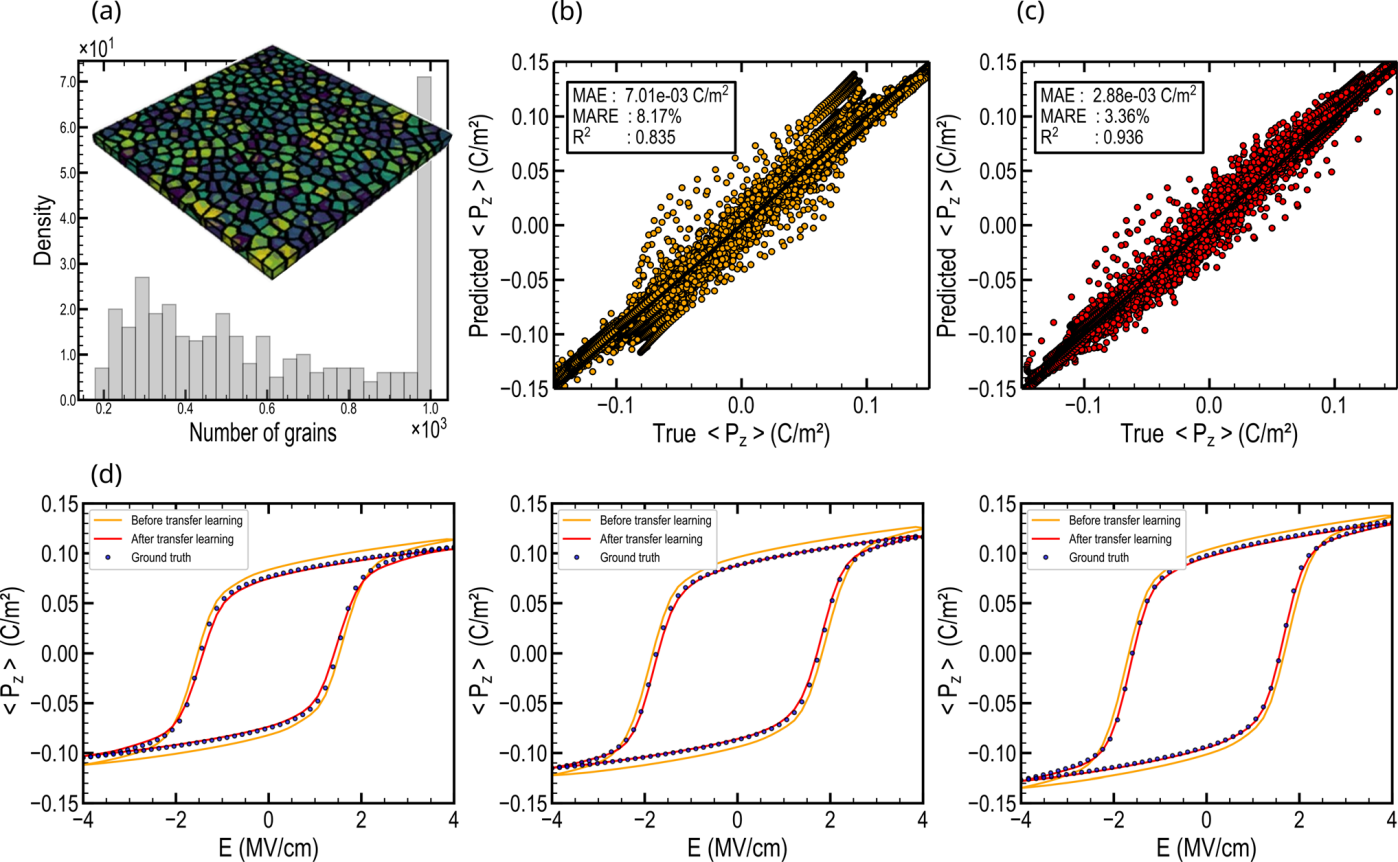
Scalability of the GNN approach to larger systems. (a) Distribution of the grain number over the 350 polycrystalline structures of 256 × 256 × 10 nm, highlighted by an illustrative example of a large Voronoi structure comprising approximately 500 ferroelectric grains. Parity plot showing the comparison between predicted and targeted out-of-plane polarization evaluated on the testing set, (b) before transfer learning and (c) after transfer learning. (d) Examples of prediction of the GNN model before and after transfer learning and the corresponding ground truth drawn from the testing set.

In this section, we explore the scalability of our framework to systems comprising up to a thousand grains. In this context, we generated 350 phase-field simulations on an 8-fold larger system (256 × 256 × 10 nm), with systems containing from 200 up to 1000 grains as shown in Fig. 8a. The testing dataset consists of 300 of the larger structures. Directly feeding the larger graphs to the pre-trained model on the previous smaller systems leads to accurate predictions, although slightly inferior in certain cases. The MARE computed on the testing dataset is 8.17%, and quantitatively, the MAE is 7.01 C m^−2^, as depicted in Fig. 8b. With a MARE below 10%, the framework reveals satisfying generalization to larger graphs by leveraging knowledge learned from the smaller systems. However, the error remains larger than what was observed on the structures employed for pre-training. Remarkably, the model achieves a considerably lower coefficient of correlation *R*^2^ = 0.835 (Fig. 8b), indicating less reliable predictions compared to classical solvers. This can be explained by the fact the scaling of the hysteresis outcome by phase-field is subjected to complex short and long-range interactions. The pre-trained GNN might struggle to accurately capture these complex interactions, leading to the slightly less accurate predictions observed in the scaled-up scenarios.

Therefore, we adopt transfer learning, a technique that leverages the knowledge gained during pre-training on a smaller system and then adjust the model on a larger system.^75–78^ To this end, we fine-tuned the pre-trained model using a limited dataset of 50 structures from the larger system (see Methods). Transfer learning yields highly accurate results, assessed on the testing dataset, with a MARE of 3.36% and a *R*^2^ coefficient of 0.936 (Fig. 8c). Qualitatively, the MAE equals 2.88 C m^−2^ and after fine-tuning, the hysteresis predicted aligns coherently with the targeted ground truth (Fig. 8d). Phase-field computational times for these larger systems increase by a factor of ∼20, requiring ∼1 hour to compute one hysteresis. Meanwhile, GNN inference time increases to 1 ms per hysteresis prediction (respectively 5 ms on CPU), resulting in an acceleration of 3.6× 10^6^ (respectively 720 000 on CPU) on large graphs.

By leveraging insights gained from pre-training on the smaller system, the model can effectively generalize to complex grain structures while accounting for increased graph size and long-range interactions. The transfer learning approach minimizes the need for extensive data from the larger system, highlighting a promising approach for efficiently up-scaling GNNs to representative polycrystalline samples.

## Discussion

In this article, we have introduced an original approach to achieve fast and accurate prediction of hysteresis in polycrystalline hafinum oxide ferroelectrics. The framework presented here incorporates graph attentional layers and an encoder-decoder configuration. By effectively capturing the electrostatic interactions between grains during the learning process, our GNN-based surrogate model has achieved remarkable performance with a MARE below 5% and a *R*^2^ score above 0.95. The model demonstrates notable flexibility, predicting a diverse array of ferroelectric hysteresis across a wide range of polycrystalline structures, grain numbers, and Landau coefficients.

To address the concern of modeling real-size HZO samples, we explored transfer learning techniques to successfully scale up the predictive capabilities of GNNs to systems containing thousands of grains. Given its accuracy and computational efficiency, our approach holds potential to serve as a surrogate differentiable model for tackling ferroelectric inverse problems. By leveraging this approach, extensive searches through the parameter space could be conducted to tailor ferroelectric materials, similar to established practices in other fields utilizing surrogate models.^42,79–83^ Calibration of Landau coefficients, which will be addressed in a future work, could be accomplished within a remarkably reduced timeframe, while eliminating the complexities associated with computational limitations when solving PDEs. Besides, a notable advantage of our GNN-based approach is its full differentiability, which sets it apart from the conventional phase-field method. This feature would allow us to address inverse problems more effectively, taking advantage of the GNN's differentiability, as has been successfully accomplished in other applications.^42,79–83^

In this research, the emphasis has been placed on refining the phase-field representation to incorporate key factors for predicting polycrystalline ferroelectric hysteresis. By augmenting the complexity of the phase-field simulations, the complexity of the GNN framework could be enhanced as well. These parameters could cover a spectrum of considerations, including global factors such as temperature and voltage ramp speed. For instance, the model could undergo extension to facilitate the prediction of the *P*(*E*) hysteresis curves, across varying temperatures. This expansion would broaden its applicability beyond the scope of the current study, which exclusively focuses on room temperature conditions. Furthermore, local parameters pertinent to individual grains and grain boundaries, such as dielectric permittivity, defect concentration, and the polar/non/polar nature of grains, could also be thoughtfully integrated. While the inclusion of each of these elements would increase the complexity of the prediction task, it would contribute to a more comprehensive and accurate representation. Importantly, these parameters could be tailored and optimized during the process of inverse design for ferroelectric hafnium oxide.

As another potential further development, our model could be adapted to phase-field modeling involving distinct electrical and mechanical boundary conditions by exploiting transfer learning. This adaptability could be achieved without necessitating an extensive retraining process, but only a few training examples. For instance, adjustments such as substituting the top electrode with an atomic force microscopy tip or applying mechanical constraints to the ferroelectric films exemplify potential modifications. Such flexibility would ensure alignment with diverse numerical and experimental set-up requirements.

## Conclusions

In summary, our ultrafast, flexible, and accurate GNN framework represents a significant advancement in the field of computational materials, particularly in predicting the ferroelectric hysteresis of polycrystalline HZO. By bridging the computational gap in phase-field simulations, our work aims to facilitate the discovery and optimization of ferroelectric materials. We anticipate that our contribution will inspire further exploration and drive meaningful advancements in ferroelectric and computational materials science.

## Experimental methods

### Phase field modeling of polycrystalline hafnium oxide

To generate the dataset, phase-field simulations were carried out according to the method described in previous studies.^26,84^ The simulations were run on a discretized grid with a size of 64 × 64 × 10, with a grid spacing of Δ*x* = Δ*y* = Δ*z* = 1 nm. The microstructure evolution of the *P*(*r*, *t*) spontaneous polarization is given by the time-dependent Landau Ginzburg (TDGL) equation:1
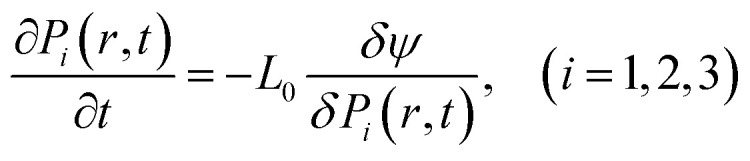
where *L*_0_ is a kinetic coefficient and *ψ* is the total free energy, which includes the different energetic contributions,^26^2
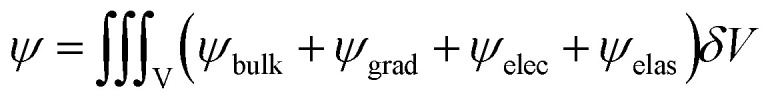


In the orthorhombic phase of HZO, the polarization is along the *c*-axis, and the bulk free energy is described by *ψ*_bulk_ = *αP*_z_^2^ + *βP*_z_^4^ + *δP*_z_^6^ where *P*_z_ is the out-of-plane polarization. The electric energy is given by 
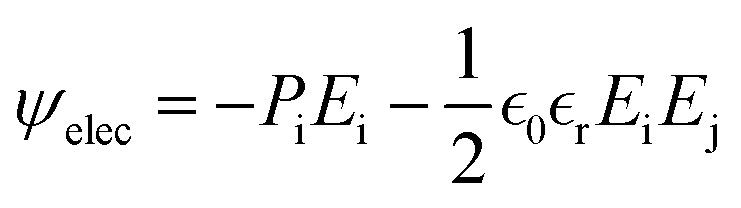
 where *E* is the electric field and *ε*_0_ and *ε*_r_ are respectively the vacuum and HZO dielectric permittivity. The elastic energy density is described by 
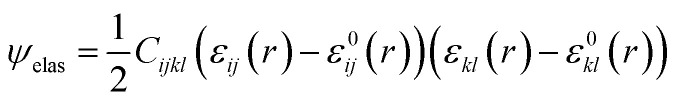
 where *C* is the elastic stiffness tensor, *ε* is the total strain and *ε*^0^ is the electrostrictive strain.^26^

The electrostatic equilibrium 
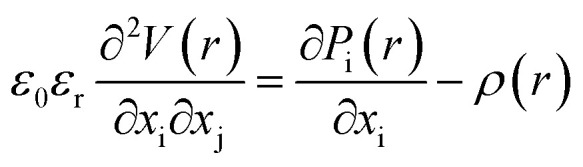
, where *V* is the electrostatic potential and *ρ* is the electric charge, is solved using the Fourier spectral method,^26^ by employing in-plane periodic boundary conditions, along with out-of-plane Dirichlet boundary conditions. Hence, we use the discrete sine transform (DST) along the *z* axis for Dirichlet boundary conditions, and discrete Fourier transforms (DFT) along the *x* and *y* axes for periodic boundary conditions. Additional details on the Fourier spectral method are available in the ESI.†

The mechanical equilibrium 
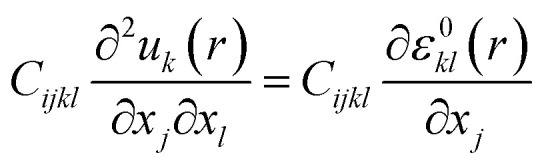
, where *u* are the mechanical displacements, is solved using thin film mechanical boundary conditions.^11^ These conditions entail a mechanically stress-free top surface and zero displacement at the bottom substrate surface, located sufficiently far from the substrate/film interface. Additionally, mechanical periodic boundary conditions are applied to the in-plane dimensions.

To modify the ferroelectric grain orientations in the structure, each ferroelectric grain was randomly assigned two angles (*θ*_1_^G^,*θ*_2_^G^) to set the orientation of its polarization axis. The transformation involves a first rotation *R̂*_*y*_(*θ*_1_^G^) of *θ*_1_^G^ around the *y*-axis, followed by a second rotation *R̂*_*z*_(*θ*_2_^G^) of *θ*_1_^G^ around the *z*-axis. The corresponding rotation matrix *R* = *R̂*_*z*_(*θ*_2_^Ĝ^) × *R̂*_*y*_(*θ*_1_^G^) was then used to compute the free energy in the local crystalline system.
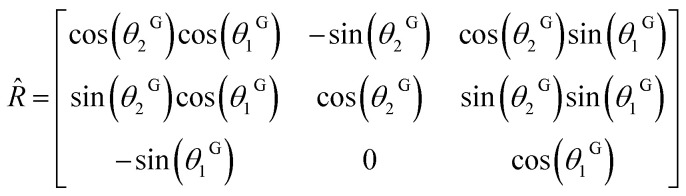


In this system, any vector *r*^L^ = (*x*^L^, *y*^L^, *z*^L^) can be obtained according to the grain's orientation, from the relation *r*^L^ = *R̂* × *r* where *r* = (*x*, *y*, *z*) is the original vector in the global system.

The HZO dielectric permittivity was chosen as *ε*_r_ = 30,^24,85^ the gradient energy coefficient *G*_110_ as *G*_110_ = 5.066 × 10^10^ C^−2^ m^4^ N.^24^ The elastic coefficients were selected with the values found in ref. 24. It is important to note that there is limited existing literature on thin films of hafnium oxide regarding these elastic and electrostrictive coefficients. The time step was taken as Δ*t* = 0.06*t*_0_ with *t*_0_ = 1/(*α*_0_*L*_0_).

Our polycrystalline structures were generated using Voronoi tesselation, containing a mix of columnar and equiaxed grains. Fig. S1† illustrates the wide diversity of polycrystalline structures that result from random centroids generation and grain size variation. We set the grain boundary thickness to 1.2 nm. The polarization within the grain boundaries was fixed to 0 C m^−2^, as in other phase-field studies involving polycrystalline grains.^17^ Further insights into the domain state evolution obtained through phase-field modeling in the presence of grain and grain boundaries are provided in the ESI.†

Ferroelectric hystereses were conducted by applying a uniformly discretized voltage ramp of 100 steps between −4 and 4 volts. To achieve this, a constant voltage was applied at each step by prescribing the voltage in the Dirichlet boundary conditions to the top electrode for a duration of 50Δ*t*, which approximately corresponds to a quasi-static regime.

Unlike previous phase-field simulations of polycrystalline hafnium oxide, which indicated that elastic energy has minimal impact on hysteresis behavior,^24^ we examined the impact of elastic energy on hysteresis. In Fig. S10,† we analyzed the changes induced by solving or no the mechanical equilibrium. As in ref. 24, we did not observe noteworthy changes in the results. Consequently, it is important to note that we do not further solve the mechanical equilibrium in this study. During the simulations, we solve for electrostatic equilibrium, enabling consideration of electrostatic interactions between grains. The motivation behind this choice is elucidated in Fig. S11,† highlighting the impact of depolarizing energy on ferroelectric hysteresis. The simulations, carried out both with and without solving electrostatic equilibrium, show a substantial alteration of the coercive field. These observations underscore the importance of solving the Poisson equation for accurately capturing the polarization charge formation within grains and at grain boundaries. As grains interact electrically, they no longer switch independently. This results in a reduced coercive field, as shown in Fig. S11.†

### Training details and evaluation metrics

Model parameters were optimized using the Adam optimizer^86^ with a batch size of 32 over 500 epochs until convergence was achieved. Detailed training history is provided in Fig. S12.† The initial learning rate was fixed at 10^−3^ and gradually reduced to 10^−6^, following an exponential decay. The Adam's default parameters were used (*β*_1_ = 0.9, *β*_2_ = 0.99 and *ε* = 10^−7^). We used the mean square error (MSE) loss function 
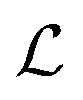
 as the training objective,3
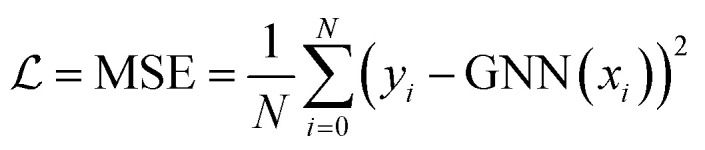
where {*x*_*i*_,*y*_*i*_}_*i* = 1_^*N*^ are the features and labels over the training dataset, and GNN(*x*_*i*_) stands for the network prediction.

All input data into the GNN framework were normalized between 0 and 1. The training was performed with an NVIDIA GeForce RTX3080 with 10 GB RAM and took approximately 15 minutes to be completed. The framework was developed in Pytorch^87^ and graph neural networks implementation was done using PyTorchGeometric^74^ library.

Regarding the transfer learning section to larger systems, a pre-trained model on the smaller systems was trained over 200 epochs with a learning rate of 10^−4^ on 50 of the larger structures.

Model performance was evaluated by reporting the macro average relative error (MARE),^39^ computed as4
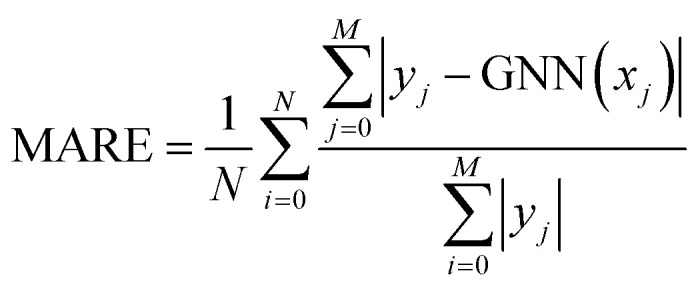
where *M* is the number of points in the hysteresis and *N* is the number of samples in the tested dataset. To report quantitative results, the mean absolute error (MAE) was also computed as5
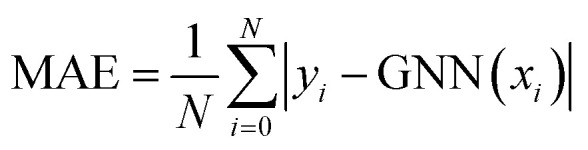


The coefficient of determination *R*^2^ was computed using the scikit-learn python library.^88^

### Graph convolutional layer

The GCNs used for the ablation study are built over massage-passing layers (MLPs) which allow learning the interaction of nodes based on the graph connectivity and the features of their neighbors. The layer-wise propagation rule used for graph convolutional layer in this paper is given by:^70^6
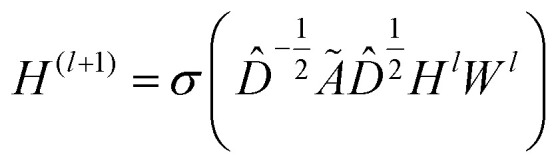
where *Ã* = *A* + *I* denotes the adjacency matrix with added self-connections, 
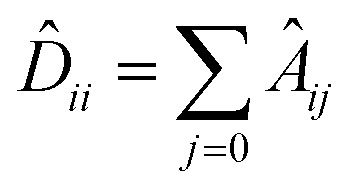
 is the diagonal degree matrix, *H*^*l*^ and *H*^(*l*+1)^ are the current and new nodes embeddings, *W*^*l*^ is a trainable weight matrix specific to the layer and *σ* an activation function.^70^

Its node-wise formulation gives the new node embedding 
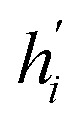
 as follows:7
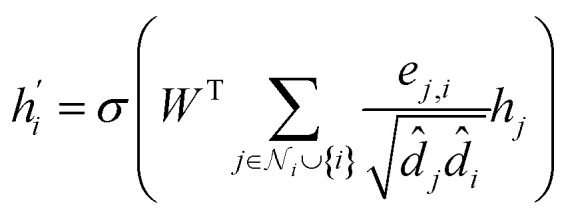
with 
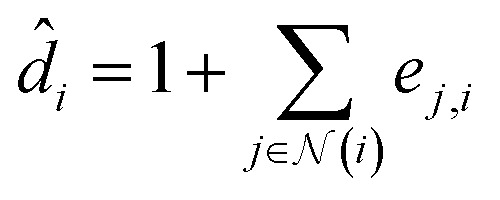
 where *e*_*j*,*i*_ denotes the edge weights between nodes *i* and *j*,^74^*W* is a trainable weight matrix and ^T^ represent transposition operation. We used the RELU activation function whose expression is given by RELU(*x*) = max(0, *x*), batch-normalization layer after each MLP and drop-out with a rate of 0.2 during training.

### Graph attentional layer

By using attention mechanisms, graph attentional layers^71^ are able to evaluate the influence of edge and node level features on the interaction of two grains. The graph attentional operator used in this paper is given by:^71^8
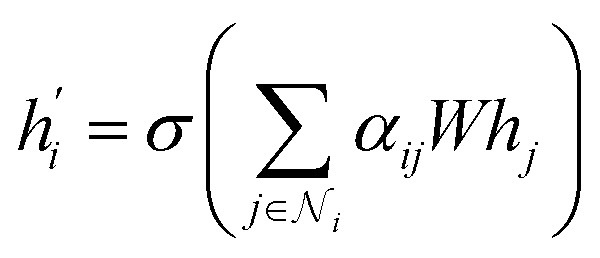
where *α*_*ij*_ is an attention coefficients between nodes *i* and *j*. It is computed as^71^9
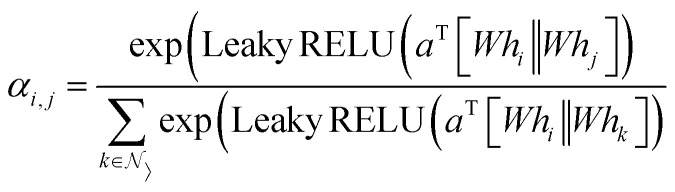
where *a* is a shared attentional mechanism computing the self-attention on the nodes, *W* is a trainable matrix. ^T^ and ‖ represent transposition and concatenation operation and LeakyRELU is the Leaky Rectified Linear Units with a slope *α* = 0.2.

### Multi-layer perceptron

Multi-layer perceptrons are dense neural networks consisting of an input layer, hidden layer(s), and a final output layer. Each layer has its own trainable weights *W* and biases *b*. The feed-forward computation in a multi-layer perceptron of a layer *i* is given by10
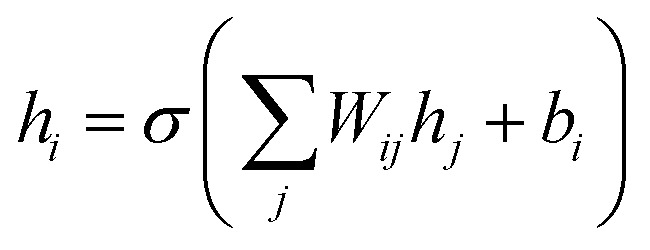
where *σ* is an activation function and *h*_*i*_ and *h*_*j*_ are respectively the value of units at layer *i* and all the related preceding layers *j*.

### Model hyperparameters

We implemented the encoder as two multi-layer perceptrons *ε*^v^ and *ε*^e^. *ε*^v^ and *ε*^e^ layers have respective sizes of [3 128 256] and [4 128 256] as the inputs node and edge features are of dimensions 3 and 4, respectively. After each layer, RELU activation and batch normalization were applied.

The decoder network *η* is a multi-layer perceptron with layers of size [256 128] where RELU activation and batch are applied after the first layer.

Message-passing layers consist of graph attentional layers^71,74^ (or graph convolutional^70,74^ layers during architecture comparison). Each MPL is followed by a RELU activation layer and a drop-out layer with the rate set to 0.2.

Node features are averaged across the node dimensions of the final graph representation using a global mean pool layer.^74^ For hysteresis prediction, the final network is an MLP with two hidden layers and an output layer of size [128 256,128 100]. We used RELU after each hidden layer and a hyperbolic tangent after the final output layer for predicting. The drop-out rate was set to 0.1. Based on the parameterization outlined in this section, the model introduced in this paper consists of 248 292 trainable parameters.

## Author contributions

K. A. L get the original idea, ran the phase-field simulations, conceived the GNN framework, performed the analysis, and wrote the manuscript. K. A. L, D. D. and B. G. worked on the development of the phase-field modeling code. All authors reviewed and discussed the manuscript.

## Conflicts of interest

The authors declare no financial or non-financial competing interests.

## Supplementary Material

NA-006-D3NA01115A-s001
