# Supplementary material for: Ultrafast and accurate prediction of polycrystalline hafnium oxide phase-field ferroelectric hysteresis using graph neural networks
Source: Nanoscale Adv. 2024 Apr 2;6(9):2350–62. doi: 10.1039/d3na01115a (PMC11059552; doi:10.1039/d3na01115a)
Supplement: NA-006-D3NA01115A-s001 [file NA-006-D3NA01115A-s001.pdf]

Electronic Supplementary Material (ESI) for Nanoscale Advances.
This journal is © The Royal Society of Chemistry 2024

Supporting Information : Ultrafast and Accurate Prediction of Polycrystalline Hafnium Oxide Phase-Field Ferroelectric Hysteresis using Graph Neural Network

ALHADA–LAHBABI Kévin [1] **DELERUYELLE Damien** [1] **GAUTIER Brice** [1]

1. Polycrystalline structures generated by Voronoi tesselation

Figure S.1 showcases examples of polycrystalline structures generated through Voronoi tessellation. These structures exhibit a mix of columnar and equiaxed grains, the arrangement of which depends on the grain sizes. We systematically varied the grain diameter within the range of 7.5 to 20 nm, resulting in structures containing between 15 to 150 grains each.

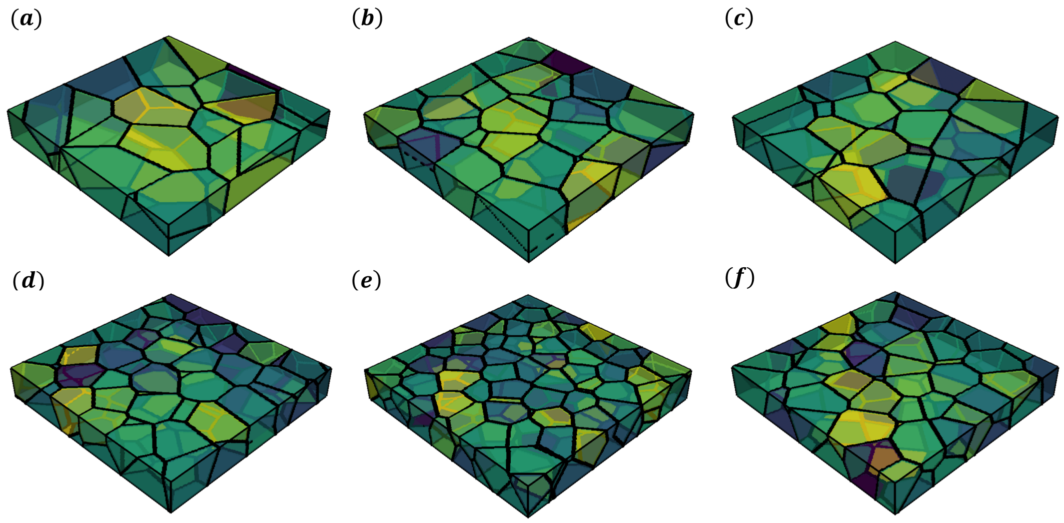

Figure S.1. **Voronoi polycrystalline structures of the generated dataset.** Voronoi structures of different grain diameters ranging from 7.5 to 20 nm. The corresponding structures contain 21 **(a)**, 37 **(b)**, 29 **(c)**, 74 **(d)**, 134 **(e)** and 58 **(f)** ferroeelectric grains. Grains are colored arbitrarily.

[1]INSA Lyon, CNRS, Ecole Centrale de Lyon, Université Claude Bernard Lyon 1, CPE Lyon, INL, UMR5270, 69621 Villeurbanne, France.. Correspondence to: ALHADA–LAHBABI Kévin <kevin.alhada-lahbabi@insa-lyon.fr>.

2. Phase-field modeling of polycrystalline hafnium oxide

The phase-field simulations carried out in this work were conducted on a discretized grid with dimensions $N_x \times N_y \times N_z$, featuring a uniform grid spacing of $\Delta x = \Delta y = \Delta z = 1$ nm. The dynamic evolution of the spontaneous polarization $\boldsymbol{P}(\boldsymbol{r}, t)$ within the microstructure is governed by the time-dependent Landau-Ginzburg (TDGL) equation:

$$\frac{\partial P_i(\boldsymbol{r}, t)}{\partial t} = -L_0 \frac{\delta \psi}{\delta P_i(\boldsymbol{r}, t)}, \quad (i = 1, 2, 3) \tag{1}$$

where L_0 represents the kinetic coefficient, and ψ denotes the total free energy, which includes the different energetic contributions, as outlined in the manuscript:

$$\psi = \int\int\int_V (\psi_{bulk} + \psi_{grad} + \psi_{elec} + \psi_{elas}) \delta V \tag{2}$$

In this investigation, polycrystalline structures were synthesized via Voronoi tessellation, comprising a mix of columnar and equiaxed grains. For all polycrystalline configurations, a grain boundary thickness of 1.2 nm was employed, aligning with the typical order of magnitude observed in experimental setups (1). An exemplary polycrystalline structure from the dataset is showcased in **Figure S.2**, representing a system of dimensions $N_x \times N_y \times N_z = 64 \times 64 \times 10$. To facilitate visualization, a structure with relatively large grains, constituting approximately 10 grains in total, was deliberately selected. The internal grain and grain boundary architectures are elucidated through 2D cross-sectional analyses conducted at $y_0 = 25$ (**Figure S.2**a), $x_0 = 50$ (**Figure S.2**b), and $z_0 = 5$ (**Figure S.2**c). Furthermore, a comprehensive 3D representation of the structure is provided in **Figure S.2**d.

To modify the orientations of ferroelectric grains within the structure, each grain underwent a random assignment of two angles (θ_1^G, θ_2^G) to define the orientation of its polarization axis. This transformation involved an initial rotation $\hat{R}_y(\theta_1^G)$ by θ_1^G around the y-axis, followed by a subsequent rotation $\hat{R}_z(\theta_2^G)$ by θ_2^G around the z-axis. The resulting rotation matrix $\hat{R} = \hat{R}_z(\theta_2^G) \times \hat{R}_y(\theta_1^G)$ was then utilized to calculate the free energy within the local crystalline system.

$$\hat{R} = \begin{bmatrix} \cos(\theta_2^G)\cos(\theta_1^G) & -\sin(\theta_2^G) & \cos(\theta_2^G)\sin(\theta_1^G) \\ \sin(\theta_2^G)\cos(\theta_1^G) & \cos(\theta_2^G) & \sin(\theta_2^G)\sin(\theta_1^G) \\ -\sin(\theta_1^G) & 0 & \cos(\theta_1^G) \end{bmatrix}$$

In this system, any vector $r^L = (x^L, y^L, z^L)$ can be obtained according to the grain's orientation, from the relation $r^L = \hat{R} \times r$ where $r = (x, y, z)$ is the original vector in the global system.

Incorporating the influence of grain boundaries into the simulation, the ferroelectric polarization within these boundaries was prescribed as zero, consistent with established studies on polycrystalline ferroelectrics (2). The polarization state within the grain boundaries is explicitly defined by the expression:

$$\boldsymbol{P}(\boldsymbol{r} = \boldsymbol{r_{GB}}, t) = \boldsymbol{0} \tag{3}$$

Here, $\boldsymbol{r} = \boldsymbol{r_{GB}}$ denotes the spatial coordinates corresponding to the locations of the grain boundaries within the polycrystalline system. Consequently, substantial depolarizing charges arise at the grain boundaries, manifesting as $\rho(\boldsymbol{r}) = -\nabla \cdot \boldsymbol{P}(\boldsymbol{r})$. This phenomenon induces a consequential depolarizing field during the resolution of the electrostatic equilibrium, significantly enhancing the electrostatic coupling between ferroelectric grains within polycrystalline thin films. While our chosen approach involves modeling grain boundaries as areas characterized by zero ferroelectric polarization, it is essential to acknowledge that alternative methodologies for depicting these boundaries have been suggested in the existing literature (3).

In the absence of specific data on the properties of grain boundaries in HZO, we opted to assign the permittivity and elastic parameters the same values as those attributed to the grains. A potential extension for handling materials with varying grain boundary properties could involve modifying the resolution approach for electrostatic and mechanical equilibrium equations to incorporate variable coefficients (4).

To illustrate the modeling of the ferroelectric structure in the presence of grains, we present in **Figure S.3** the domain structure resulting from a simulation of domain formation. In this simulation, polarization was randomly initialized at each

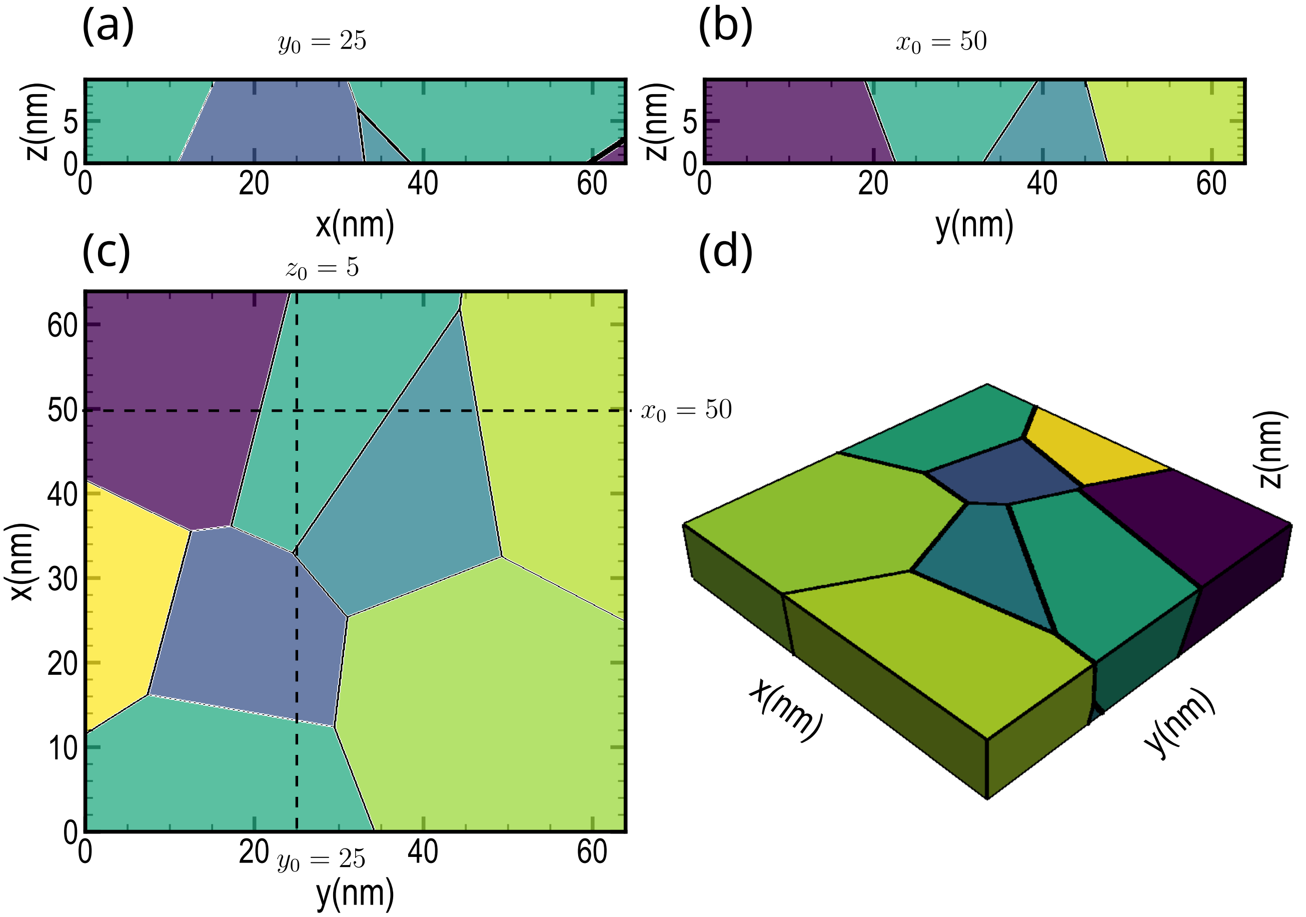

Figure S.2. **Illustration of a 3D polycrystalline structure achieved through Voronoi tessellation.** Detailed 2D cross-sectional insights of the grain distribution are given at specific planes: **(a)** $y_0 = 25$, **(b)** $x_0 = 50$, and **(c)** $z_0 = 5$. Additionally, **(d)** provides a global 3D depiction of the polycrystalline system. Grains are arbitrarily colored for clarity in visualization.

grid point within the films, and the domains were allowed to evolve freely until reaching equilibrium. Notably, no voltages were applied during this process. Detailed 2D cross-sections of the ferroelectric polarization are presented at specific planes: $y_0 = 25$ (**Figure S.3**a), $x_0 = 50$ (**Figure S.3**b), and $z_0 = 5$ (**Figure S.3**c). In all these 2D cross-sections, the colorbar corresponds to the out-of-plane component $\mathcal{P}_z$ value, while the 2D arrows provide insights into the polarization orientation within each grain. A 3D representation illustrating the magnitude of the out-of-plane polarization is also presented in **Figure S.3**d. Furthermore, a 3D representation of the polarization vector is provided in **Figure S.4**, highlighting distinct domains within each grain along with the polarization orientation in the polycrystalline arrangement. Those views reveal the organization of polarization into multiple domains within each grain, separated by domain walls. The orientation of these domains within each grain is contingent upon the crystalline orientation of the grain. Additionally, at each grain boundary, the polarization is observed to be fixed at **0**.

Furthermore, the dynamic evolution of the internal domain structure during a typical ferroelectric hysteresis is depicted in **Figure S.5**. The representation of the domain structure is provided through 2D cross-sections in the plane $x_0 = 50$, at various points along the $P_z(E)$ curves in **Figure S.5**a-d. This visualization elucidates the switching process occurring within each grain and accentuates the global electrical coupling between ferroelectric grains. Notably, depending on the grain orientation, the polarization tends to switch at different moments, contingent on its interaction with the vertically applied electric field.

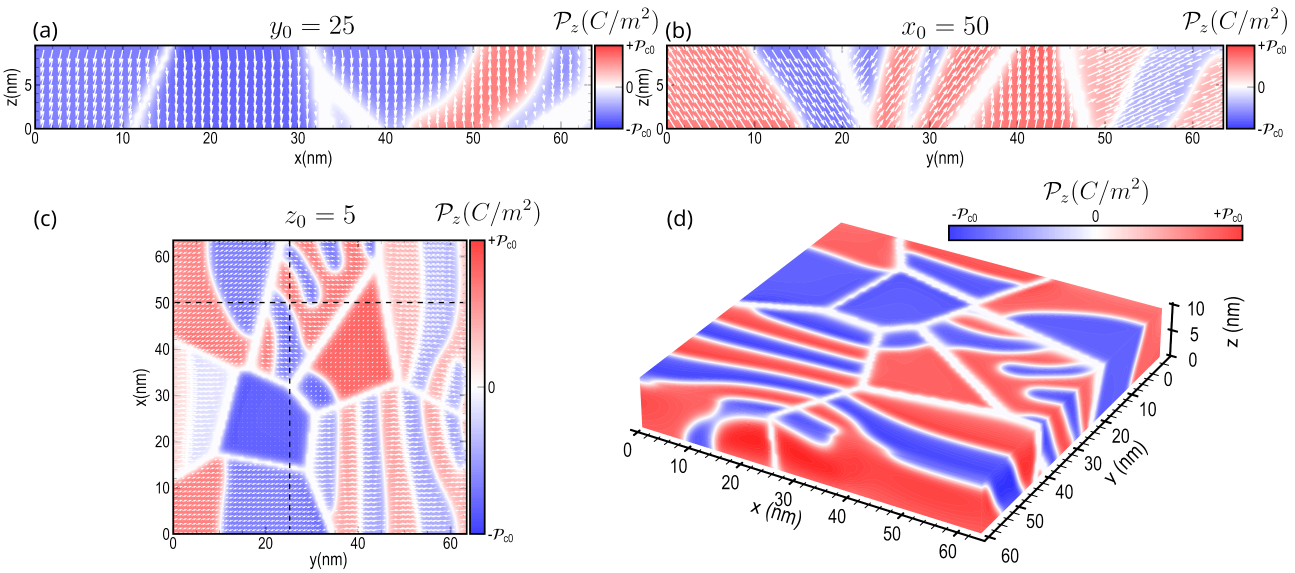

Figure S.3. **Illustration of the 3D Ferroelectric Domain State in a Polycrystalline Structure.** Detailed 2D cross-sectional insights into the ferroelectric polarization are presented at specific planes: **(a)** $y_0 = 25$, **(b)** $x_0 = 50$, and **(c)** $z_0 = 5$. The colorbar represents the out-of-plane polarization $\mathcal{P}_z$ values, while the 2D arrows denote the polarization orientation within each cross-section. **(d)** A 3D depiction of the domain state, emphasizing the intensity of $\mathcal{P}_z$.

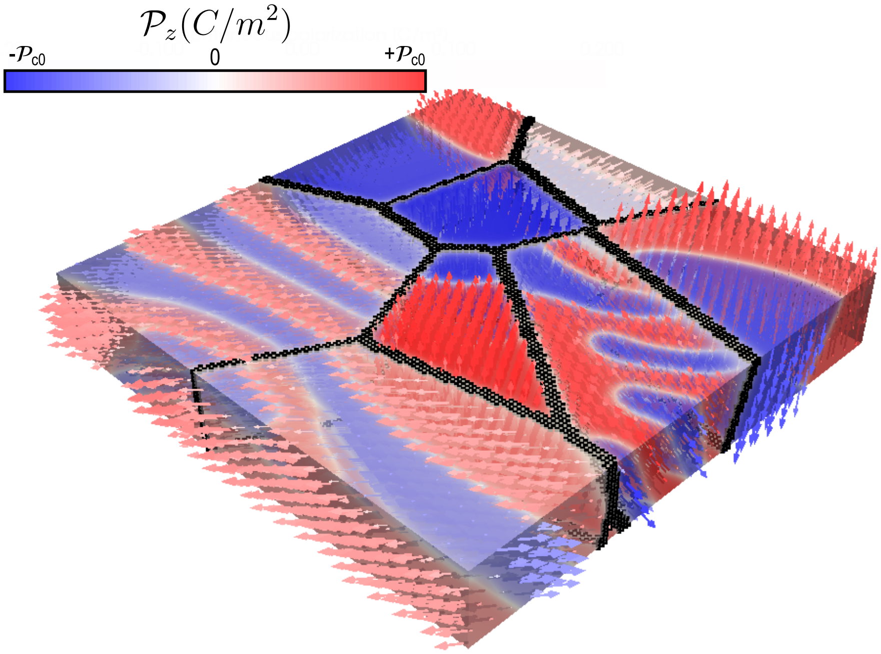

Figure S.4. **Illustration of the 3D Ferroelectric Domain State in a Polycrystalline Structure.** Comprehensive 3D visualization of the polarization domain state. The colorbar corresponds to the out-of-plane polarization $\mathcal{P}_z$ values, while the 3D arrows indicate the polarization orientation within each ferroelectric grain.

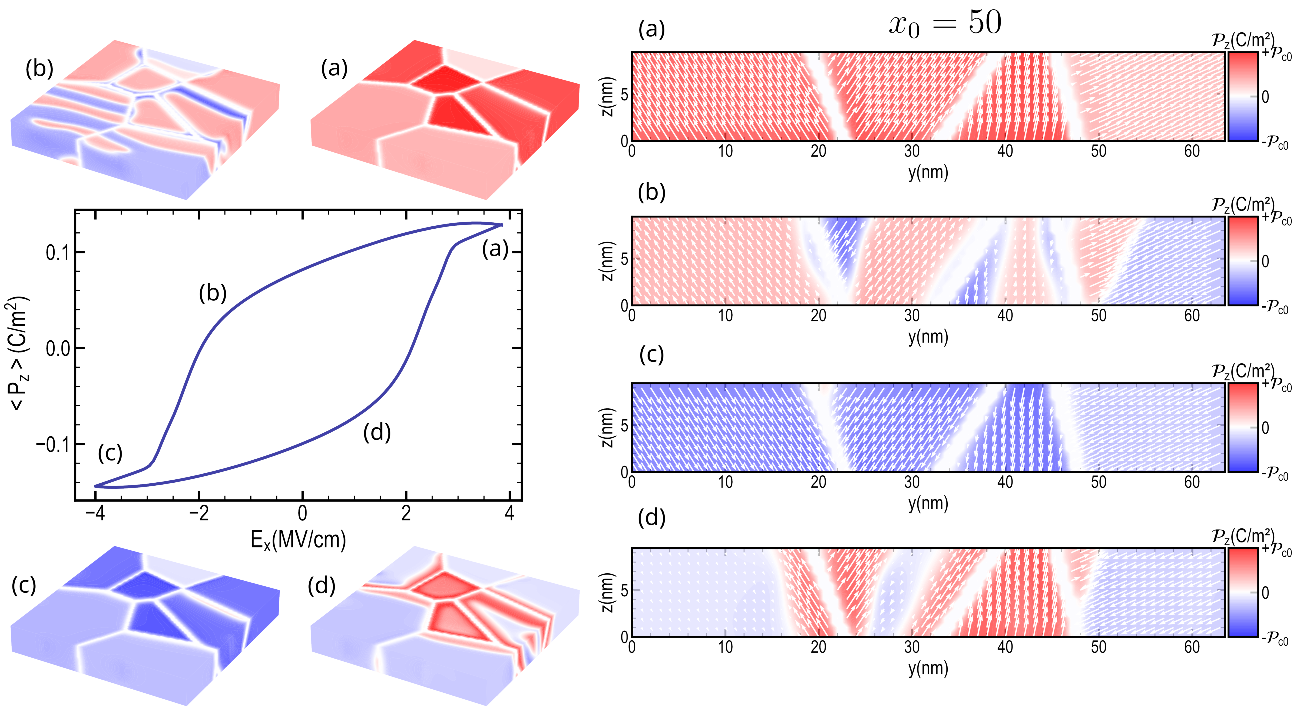

***Figure S.5*.** **Illustration of the Domain State Evolution During a Ferroelectric Hysteresis.** $P_z(E)$ curve obtained through phase-field simulations depicting the electrical hysteresis over a 10-nm-thick HZO thin film. **(a-d)** 2D cross-sections illustrating the evolution of the polarization state across the hysteresis in the plane $x_0 = 50$. The colorbar represents the intensity of the P_z component, while the 2D arrows depict the ferroelectric polarization vectors at each grid point.

Theoritical range of remanent polarization and coercive field covered by the Landau coefficients in the generated dataset

Figure S.6 represents the theoretical curve characterizing the hystereses according to Landau-Ginzburg theory, as represented in other phase-field works(5). For 1000 sets of Landau coefficients from the dataset, the theoretical evolution of the spontaneous polarization with the electric field,

$$E = \alpha P + \beta P^3 + \delta P^5 \tag{4}$$

was plotted for electric fields varying from -4 to 4 MV/cm. We can observe a wide diversity of remanent polarizations, saturation polarization, coercive field, and hystereses shapes. The target values of remanent polarization 0.15 C/m^2 and coercive field 1 MV/cm we find in our experimental measurements are well covered. Thus, the GNN could be capable of handling the range of experimental data in a future perspective for calibrating the Landau coefficient.

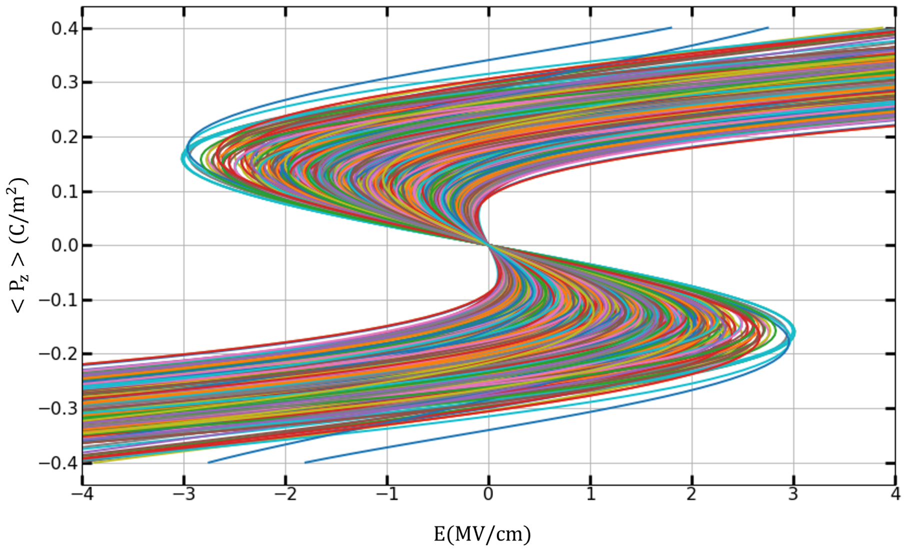

Figure S.6. **Spontaneous polarization P versus electric field E for 1000 sets of Landau coefficients from the dataset.** Theoretical relation between P and E from the Landau-Ginzburg theory. The sets of Landau coefficients taken in this study cover a broad range of remanent polarization and coercive fields around the target values of 15 C/m^2 and 1 MV/cm.

3. Diversity of the ferroelectric hystereses in the generated dataset

Figure S.7 portrays the diversity of ferroelectric hysteresis observed in this study. Diverse shapes, polariztion saturation levels, remanent polarization values, and coercive fields are observed in the hysteresis curves. This variety can be attributed to the wide range of Landau coefficients and polycrystalline structures explored in the study, emphasizing their significant roles in shaping the hysteresis diversity in the dataset.

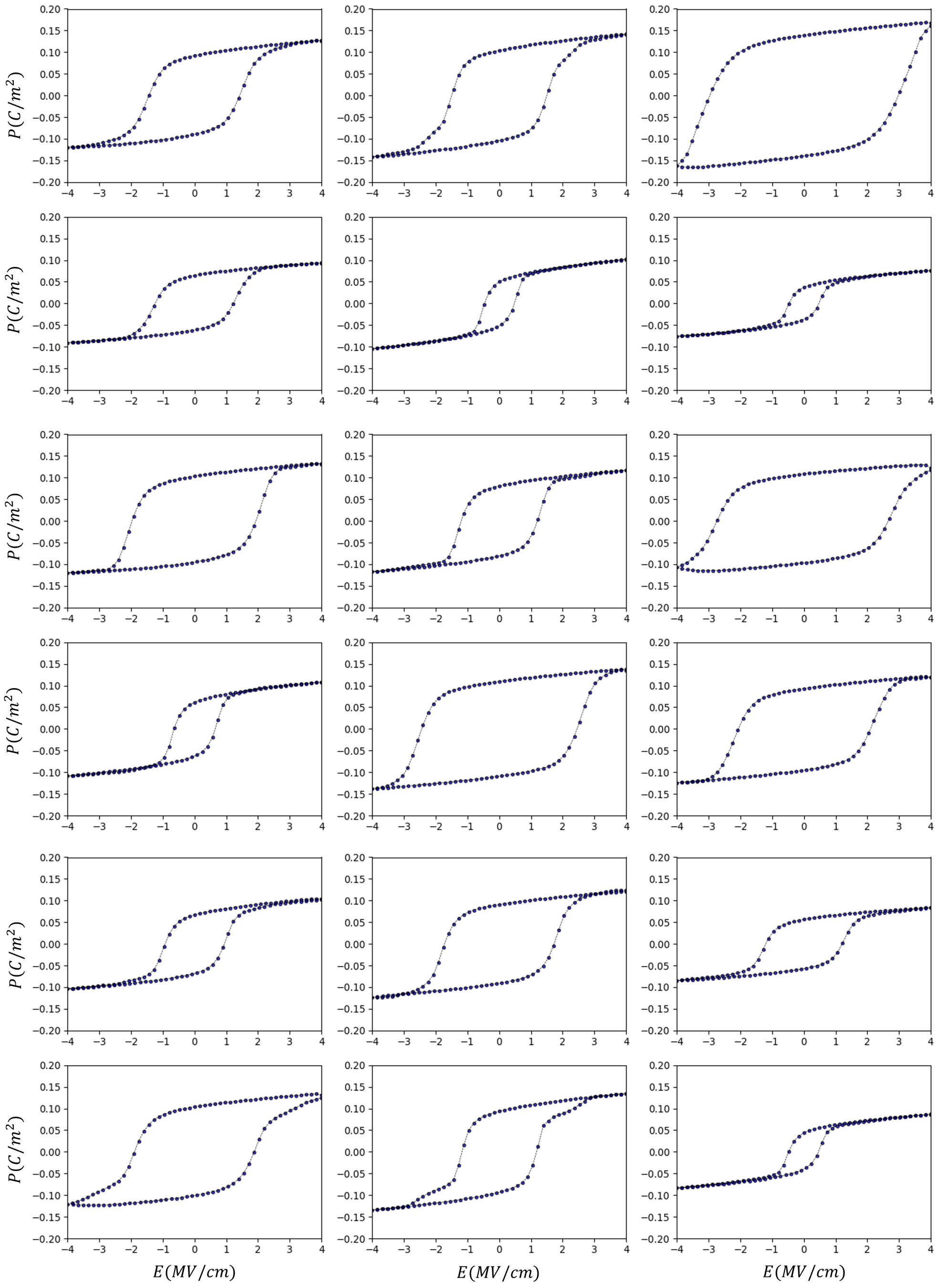

***Figure S.7*. Ferroelectric hystereses in the generated dataset computed using phase-field modeling.** Example of 18 hystereses, illustrating the diversity of shape, remanent polarization, saturation polarization, and coercive field in the dataset.

4. Influence of the Landau coefficient on the ferroelectric hystereses

Figure S.8 demonstrates the impact of the Landau coefficients on ferroelectric hysteresis. Throughout all simulations, the crystalline structure remained consistent. The Landau coefficients contribute significantly to the shape and characteristic values of the hysteresis, as displayed in **Figure S.8**.

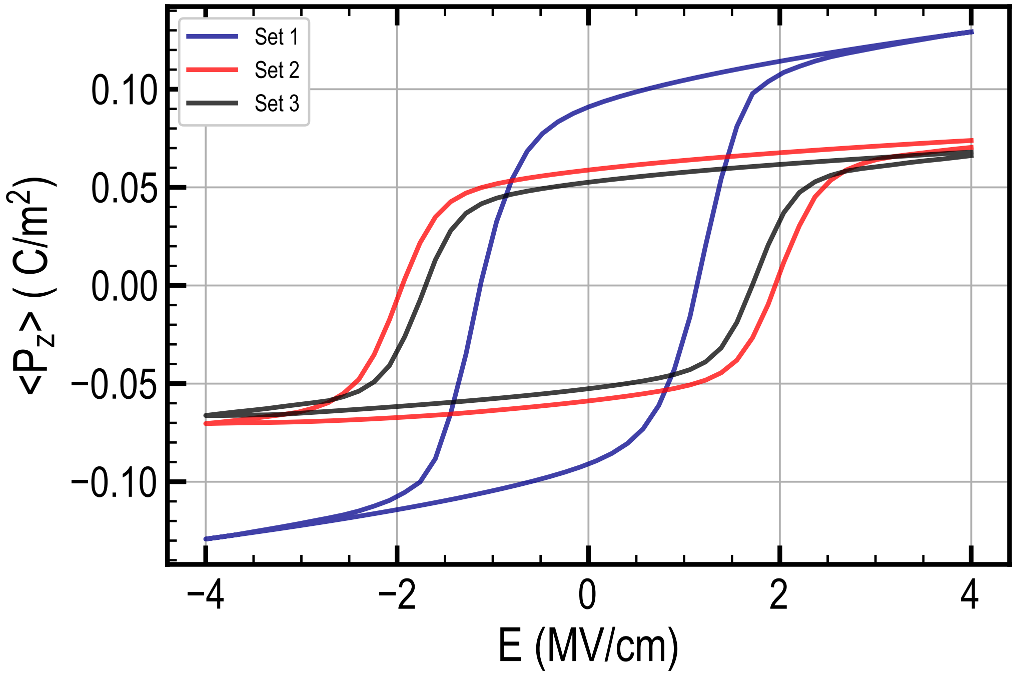

Figure S.8. **Influence of the Landau coefficients on the ferroelectric hystereses.** Three hystereses with different sets of Landau are performed using the same polycrystalline structure. Other simulation parameters are left unchanged. The three sets ($\alpha = -1.59 \times 10^9$ $C^{-2}m^2N$, $\beta = 3.34 \times 10^{10}$ $C^{-4}m^6N$, $\delta = -5.3 \times 10^{10}$ $Cm^{-6}m^{10}N$) (set 1), ($\alpha = -3.79 \times 10^9$ $C^{-2}m^2N$, $\beta = 1.81 \times 10^{10}$ $C^{-4}m^6N$, $\delta = -9.4 \times 10^{10}$ $Cm^{-6}m^{10}N$) (set 2) and ($\alpha = -3.64 \times 10^9$ $C^{-2}m^2N$, $\beta = 2.22 \times 10^{10}$ $C^{-4}m^6N$, $\delta = -9.37 \times 10^{10}$ $Cm^{-6}m^{10}N$) (set 3) lead to modification of the hystereses.

The three sets were chosen as ($\alpha = -1.59 \times 10^9$ $C^{-2}m^2N$, $\beta = 3.34 \times 10^{10}$ $C^{-4}m^6N$, $\delta = -5.3 \times 10^{10}$ $Cm^{-6}m^{10}N$) (set 1), ($\alpha = -3.79 \times 10^9$ $C^{-2}m^2N$, $\beta = 1.81 \times 10^{10}$ $C^{-4}m^6N$, $\delta = -9.4 \times 10^{10}$ $Cm^{-6}m^{10}N$) (set 2) and ($\alpha = -3.64 \times 10^9$ $C^{-2}m^2N$, $\beta = 2.22 \times 10^{10}$ $C^{-4}m^6N$, $\delta = -9.37 \times 10^{10}$ $Cm^{-6}m^{10}N$) (set 3).

5. Influence of the polycrystalline structure on the ferroelectric hystereses

Figure S.9 depicts the impact of the polycrystalline structure on ferroelectric hysteresis. Throughout all simulations, the Landau coefficients remained constant. Varied grain sizes, orientations, and random grain centroids were employed in the simulations. The polycrystalline grain structure significantly influences ferroelectric hysteresis, playing a pivotal role in determining coercive field and remanent polarization values, as demonstrated in **Figure S.9**.

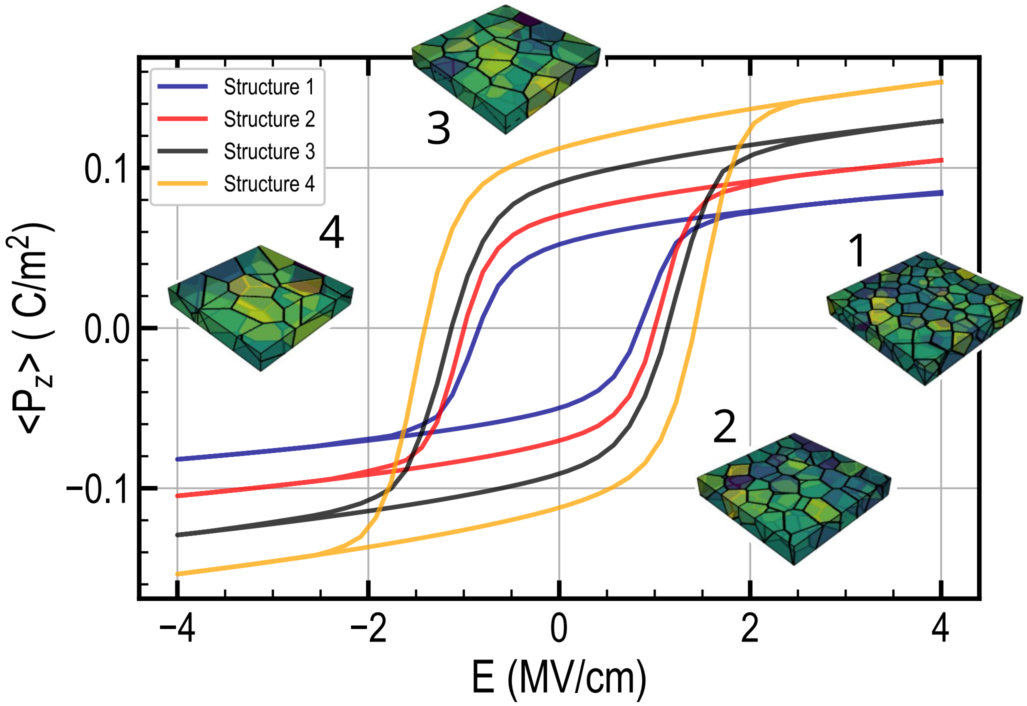

Figure S.9. **Influence of the polycrystalline structure on the ferroelectric hystereses.** Hystereses with different Voronoi structures generated are performed using the same set of Landau coefficients. Other simulation parameters are left unchanged. The four Voronoi structures used are displayed along with the hystereses.

6. Influence of the elastic energy on the ferroelectric hystereses

Figure S.10 illustrates the impact of elastic energy on ferroelectric hysteresis. The simulations maintained consistent Landau coefficients and polycrystalline structure. Notably, the influence of elastic energy proves negligible in polycrystalline hafnium oxide, corroborated by findings in Ref.6. Consequently, akin to the methodology in Ref.6, the mechanical equilibrium is not solved during the phase-field simulation for dataset generation.

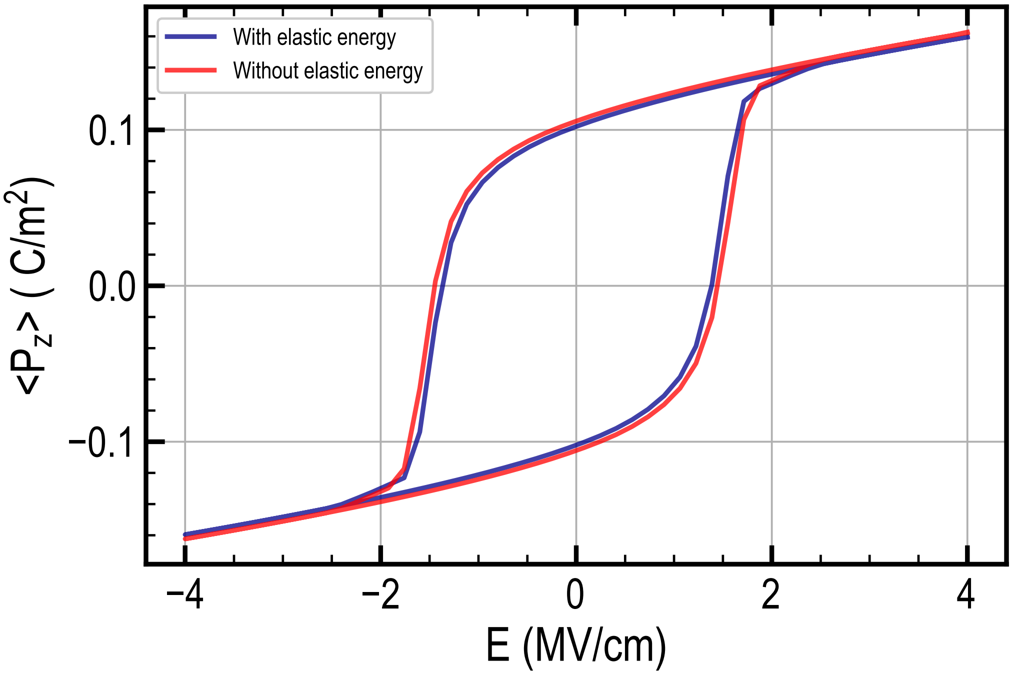

***Figure S.10*. Influence of the elastic energy on the ferroelectric.** Hystereses with and without solving the mechanical equilibrium are conducted. Simulations with and without solving the mechanical equilibrium exhibit close alignment.

7. Influence of the depolarizing field on the ferroelectric hystereses

Figure S.11 illustrates the influence of depolarizing energy on ferroelectric hysteresis. Throughout the simulations, the Landau coefficients and polycrystalline structure remained constant. To scrutinize the impact of the depolarizing field, we excluded the polarization charge term $-\nabla \cdot \boldsymbol{P}$ from the Poisson equation. In this scenario, the electric field is solely determined by the applied voltage, rendering grains electrically non-interacting. Consequently, each grain undergoes independent switching based on its orientation and coercive field.
However, reintroducing the polarization charge leads to electrical interactions between grains, altering the independent switching behavior. Polarization switches within grains can generate a charge that influences the switching of neighboring grains. This phenomenon results in a reduction of global coercive fields, as depicted in **Figure S.11**. Thus, the simulations conducted in this study incorporate the depolarizing field for a comprehensive understanding.

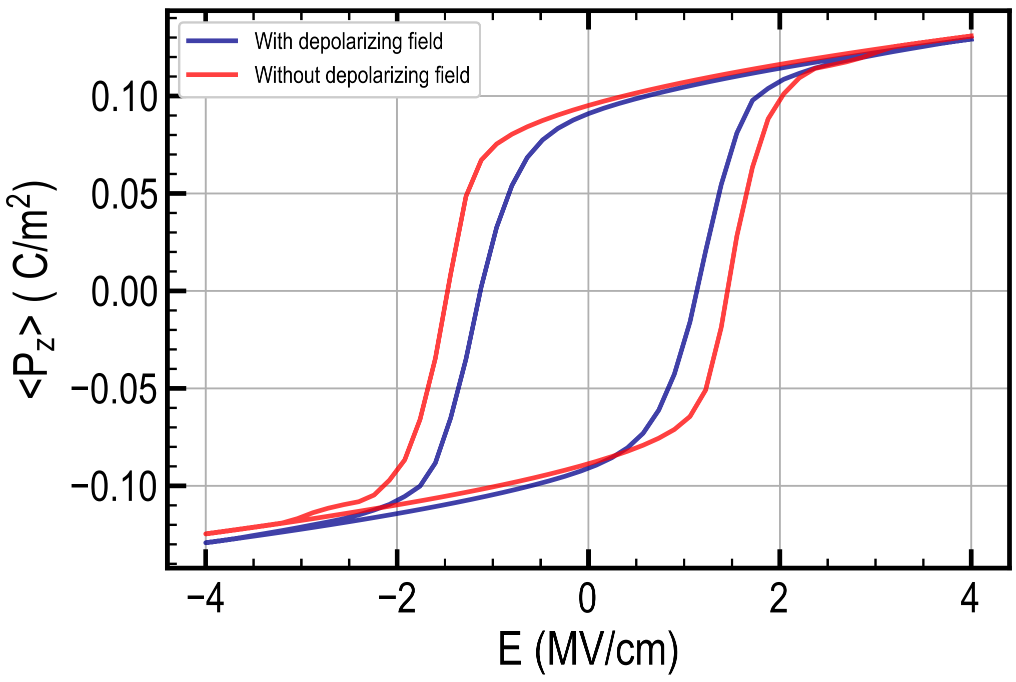

Figure S.11. **Influence of the depolarizing energy on the ferroelectric hystereses.** Hystereses with and without accounting for the polarization charge terms in the Poisson equation are conducted. Electrostatic interactions significantly modify the switching dynamics. The grain interactions result in a reduction of the coercive field.

8. Training history

Figure S.12 shows the GNN model training history. The training loss is calculated on the 3150 samples of the training dataset, and a validation loss on the 350 structures of the validation dataset. In training, the model hyperparameters were optimized to minimize the mean squared error loss function. As training progresses and validation increases, both decrease and converge.

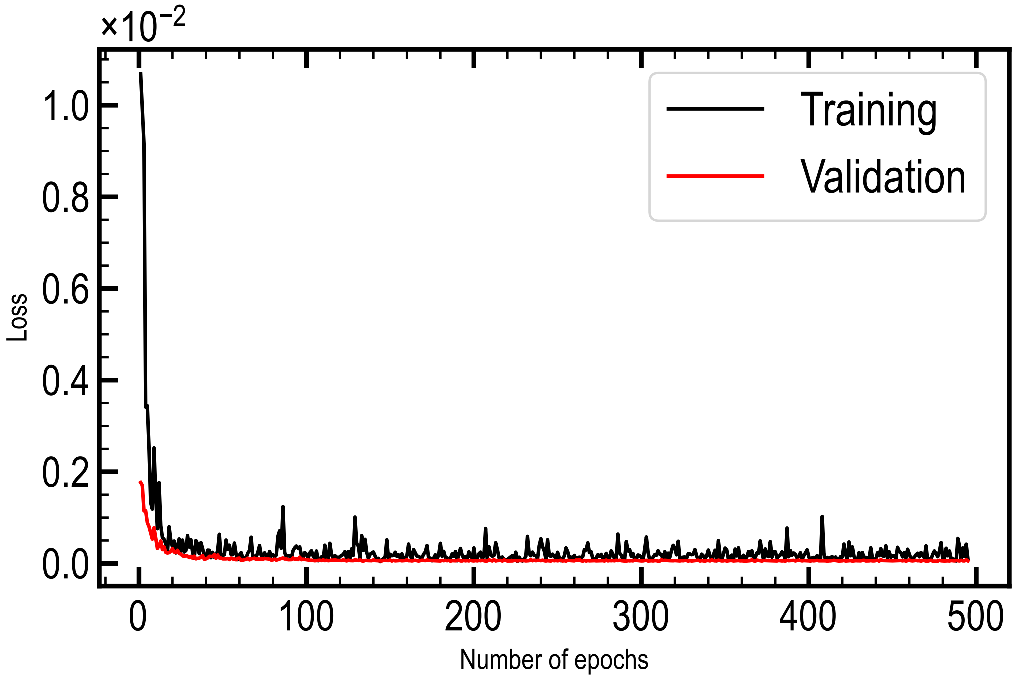

Figure S.12. **Training history of the model.** The evolution of the training and validation losses are reported during training. The model was trained for 500 epochs.

9. Spectral method for the electrostatic equilibrium

In this study, we employed the Fourier spectral method to address the electrostatic equilibrium, building upon the methodology elucidated in a previous work (7). The electrostatic equilibrium in real space is expressed by the equation:

$$\Delta V(\mathbf{r}) = f(\mathbf{r}), \tag{5}$$

where $V(\mathbf{r})$ denotes the electrostatic potential, and $f(\mathbf{r})$ signifies the right-hand side of the Poisson equation.

The solution in Fourier space, represented by the wavenumber vector $\mathbf{k} = (k_x, k_y, k_z)$, is given by the equation:

$$\tilde{V}_k(\mathbf{k}) = -G(\mathbf{k})\tilde{f}(\mathbf{k}), \tag{6}$$

where $G^{-1}(\mathbf{k}) = k_x^2 + k_y^2 + k_z^2$, and $\tilde{V}_k(\mathbf{k})$ and $\tilde{f}(\mathbf{k})$ are the Fourier transforms of the electrostatic potential and the right-hand side, respectively. The solution can be recovered in real space by performing inverse Fourier transforms.

Periodic boundary conditions are applied along the in-plane directions, leading to the use of discrete Fourier transforms (DFT) along $\mathbf{x}$ and $\mathbf{y}$, while Dirichlet boundary conditions are applied along the out-of-plane direction, resulting in a the application of the discrete sine transform (DST) along $\mathbf{z}$.

The DST is given by

$$\tilde{f}_k^{DST} = 2\sum_{n=0}^{N-1} f_n \sin\left(\frac{\pi(k+1)(n+1)}{N+1}\right), \tag{7}$$

whereas the DFT can be obtained through

$$\tilde{f}_k^{DFT} = \sum_{n=0}^{N-1} f_n e^{\frac{-i2\pi kn}{N}}, \tag{8}$$

Here, $\tilde{f}_k$ represents the spectral coefficient in Fourier space, and f_n denotes the discrete values of the electrostatic potential in real space along the considered axis. Hence, inverse discrete sine transform (IDST) and inverse discrete Fourier transforms (IDFT) were applied along the respective axes to reconstruct the solution in real space from the spectral coefficients $\tilde{V}_k(\mathbf{k})$.

The IDST is given by

$$f_n = \frac{1}{N+1}\sum_{k=0}^{N-1} \tilde{f}_k \sin\left(\frac{\pi(k+1)(n+1)}{N+1}\right), \tag{9}$$

whereas the IDFT can be obtained through

$$f_n = \frac{1}{N}\sum_{k=0}^{N-1} \tilde{f}_k e^{\frac{i2\pi nk}{N}} \tag{10}$$

While the DST has been utilized to address the Dirichlet boundary conditions along the vertical axis in this study, it is imperative to note that various spectral methods are available for such applications. As an illustrative alternative, the Chebyshev collocation method can be effectively employed to handle non-periodic boundary conditions in the resolution of partial differential equations within the context of ferroelectric phase-field modeling (8; 9).

References

[1] M. Lederer, T. Kämpfe, R. Olivo, D. Lehninger, C. Mart, S. Kirbach, T. Ali, P. Polakowski, L. Roy and K. Seidel, *Applied Physics Letters*, 2019, **115**, 22.

[2] J. Wang, W. Shu, T. Shimada, T. Kitamura and T. Zhang, *Acta Mater.*, 2013, **61**, 6037–6049.

[3] J. Wang, W. Shu, T. Shimada, T. Kitamura and T. Zhang, *Acta Mater.*, 2013, **61**, 6037–6049.

[4] J. J. Wang, X. Q. Ma, Q. Li, J. Britson and L. Q. Chen, *Acta Mater.*, 2013, **61**, 7591–7603.

[5] A. K. Saha, K. Ni, S. Dutta, S. Datta and S. Gupta, *Appl. Phys. Lett.*, 2019, **114**, 1–5.

[6] S. Sugathan, K. Thekkepat, S. Bandyopadhyay, J. Kim and P. R. Cha, *Nanoscale*, 2022, **14**, 14997–15009.

[7] K. Alhada-Lahbabi, D. Deleruyelle and B. Gautier, *ACS Applied Electronic Materials*, 0, **0**, null.

[8] Y. Cao, J. Shen, C. A. Randall and L. Q. Chen, *Applied Physics Letters*, 2014, **104**, 182905.

[9] Y. Cao, S. Bhattacharya, J. Shen, C. A. Randall and L. Q. Chen, *Journal of Applied Physics*, 2013, **114**, 224102.
